# Tree recruitment is determined by stand structure and shade tolerance with uncertain role of climate and water relations

**DOI:** 10.1002/ece3.7984

**Published:** 2021-08-19

**Authors:** Yannek Käber, Peter Meyer, Jonas Stillhard, Emiel De Lombaerde, Jürgen Zell, Golo Stadelmann, Harald Bugmann, Christof Bigler

**Affiliations:** ^1^ Forest Ecology Department of Environmental Systems Science Institute of Terrestrial Ecosystems ETH Zurich Zurich Switzerland; ^2^ Department Forest Nature Conservation Northwest German Forest Research Institute Münden Germany; ^3^ Forest Resources and Management Swiss Federal Research Institute for Forest, Snow and Landscape Research WSL Birmensdorf Switzerland; ^4^ Forest & Nature Lab Department of Environment Faculty of Bioscience Engineering Ghent University Ghent Belgium

**Keywords:** drought tolerance, forest ecosystems, forest reserves, national forest inventories, plant population and community dynamics, shade tolerance, tree recruitment, tree regeneration

## Abstract

1. Tree regeneration is a key process for long‐term forest dynamics, determining changes in species composition and shaping successional trajectories. While tree regeneration is a highly stochastic process, tree regeneration studies often cover narrow environmental gradients only, focusing on specific forest types or species in distinct regions. Thus, the larger‐scale effects of temperature, water availability, and stand structure on tree regeneration are poorly understood.
2. We investigated these effects in respect of tree recruitment (in‐growth) along wide environmental gradients using forest inventory data from Flanders (Belgium), northwestern Germany, and Switzerland covering more than 40 tree species. We employed generalized linear mixed models to capture the abundance of tree recruitment in response to basal area, stem density, shade casting ability of a forest stand as well as site‐specific degree‐day sum (temperature), water balance, and plant‐available water holding capacity. We grouped tree species to facilitate comparisons between species with different levels of tolerance to shade and drought.
3. Basal area and shade casting ability of the overstory had generally a negative impact on tree recruitment, but the effects differed between levels of shade tolerance of tree recruitment in all study regions. Recruitment rates of very shade‐tolerant species were positively affected by shade casting ability. Stem density and summer warmth (degree‐day sum) had similar effects on all tree species and successional strategies. Water‐related variables revealed a high degree of uncertainty and did not allow for general conclusions. All variables had similar effects independent of the varying diameter thresholds for tree recruitment in the different data sets.
4. *Synthesis:* Shade tolerance and stand structure are the main drivers of tree recruitment along wide environmental gradients in temperate forests. Higher temperature generally increases tree recruitment rates, but the role of water relations and drought tolerance remains uncertain for tree recruitment on cross‐regional scales.

## Graphical abstract

This study presents an analysis of tree recruitment in different regions with large environmental gradients. We show that stand structure, shade tolerance. And temperature are main drivers of tree recruitment. Furthermore, the effect of water relations is subject to a high degree of uncertainty.

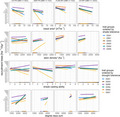

## 1 INTRODUCTION

Forest succession is defined as the shift in species composition and vegetation physiognomy over time at the level of a site, assuming that climatic conditions are constant and no major disturbance events occur (Finegan, 1984). Current climate change alters the growing conditions of trees at every spatial scale (IPCC, 2014). Thus, the probability of shifts in tree species composition in forest ecosystems increases (Boisvert‐Marsh et al., 2019; Ibáñez et al., 2008; Kroiss & HilleRisLambers, 2015). Successional trajectories that shape the structure and composition of future forest ecosystems are, along with mortality and tree growth, largely determined by tree regeneration (Fisher et al., 2018; Huber et al., 2018). However, our quantitative understanding of the main drivers of tree regeneration is limited, particularly under climate change. Thus, we must consider the interaction of successional properties of tree species and environmental factors. Furthermore, we need to search for general patterns of tree regeneration that help us to generate a better understanding of these highly stochastic processes under changing climate. Thus, studying tree regeneration on cross‐regional scales with observational data is of key importance.

Tree regeneration can be decomposed into several subprocesses, starting with seed production followed by seed dispersal, seed storage, germination, establishment of seedlings, growth of seedlings and saplings, and finally the recruitment of small trees that exceed a certain measurement threshold (cf. Price et al., 2001). Many studies have demonstrated the complexity of tree regeneration and the multidimensionality of the driving factors (Canham & Murphy, 2016, 2017; Collet & Chenost, 2006; Frei et al., 2018; Kramer et al., 2014; Kupferschmid et al., 2019). Temperature modulates the occurrence of tree regeneration to some extent. Canham and Murphy (2016, 2017) identified species‐specific responses to temperature and precipitation in eastern North America, but no general patterns among species for the survival of saplings could be detected. Collet and Chenost (2006) show that light availability is an important driver of seedling growth. Furthermore, they identified species‐specific responses to temperature and precipitation, but no general patterns among species. Kramer et al. (2014) demonstrate the importance of disturbances for tree regeneration, and Kupferschmid et al. (2019) showed that browsing drastically affects regeneration success. Frei et al. (2018) analyzed biotic and abiotic drivers of seedling mortality at the lower and upper ends of an elevational gradient and found that biotic factors are limiting at lower elevations and abiotic factors are limiting at higher elevation. This finding is also supported by extensive studies on an elevational gradient in northwestern USA (Ettinger et al., 2011; Ettinger & HilleRisLambers, 2013, 2017). Consequently, the variety of driving factors and their interactions renders tree regeneration a highly stochastic process, and predicting this process along large environmental gradients is challenging (cf. Clark et al., 1999; Lett & Dorrepaal, 2018; Price et al., 2001; Schupp, 1995; Shoemaker et al., 2020).

Most recent studies on the relationship between environmental drivers and tree regeneration compared tree species based on a range of traits that relate to different processes in complex forest ecosystems (Grime, 2006; Grubb, 1998; Lortie et al., 2004). Functional traits are assigned to tree species (Kattge et al., 2020), either by arranging them along a continuous gradient (Lai et al., 2020; Seagle & Liang, 2001) or by classifying them in groups of similar species (Halpern, 1989; Rüger et al., 2020). Thus, various studies showed that plant traits related to specific processes (e.g., shade tolerance or drought tolerance) can be useful for studying forest ecosystem dynamics and at least to some extent tree regeneration (Rüger et al., 2020; Walters & Reich, 2000). Specifically, Rüger et al. (2020) showed that shade tolerance and trade‐offs between fast‐growing and slow‐growing tree species are important drivers of tree recruitment in tropical forest ecosystems. Walters and Reich (2000) found that shade tolerance is more important for tree growth and survival of older seedlings than nitrogen supply or seedling mass by comparing early tree growth among species with different levels of shade tolerance.

While the term tree regeneration comprises multiple subprocesses, tree recruitment includes trees that exceed a given diameter at breast height (DBH) threshold for the first time in a defined time interval, also called “in‐growth” in forest science. Tree recruitment can thus serve as a proxy for successful regeneration. Tree recruitment and its relationship to the environment and stand structure have been studied for various species in different regions (Klopcic et al., 2012, 2014; Mugasha et al., 2017; Vanclay, 1992; Yang & Huang, 2015; Zell et al., 2019). All these studies on tree recruitment showed that measures of stand density (e.g., basal area or mean diameter at breast height) are important drivers of tree recruitment in temperate forest ecosystems. Compared to small‐scale experimental studies (Frei et al., 2018; Kroiss & HilleRisLambers, 2015), which for logistical reasons usually are restricted to a few sites, observational studies on tree recruitment allow for more general analyses over larger environmental gradients. Nevertheless, comparisons among these studies are difficult as they often use different analytical approaches, and assessment methods are restricted to certain regions and focus on different species. These difficulties result in three major limitations.

First, due to different analytical approaches, statistical effects of the environment and stand structure on tree recruitment cannot be compared across studies. For example, basal area seems to be negatively correlated with tree recruitment in most studies (Klopcic et al., 2012; Mathys et al., 2021; Vanclay, 1992; Zell et al., 2019) which indicates that general patterns exist. However, general statements regarding species‐specific differences in the relationship between stand structure, the environment, and tree recruitment across regions are impeded by the heterogeneity in explanatory variables and models between studies.

Second, because individual study regions differ substantially in site conditions, management regimes, and the extent of environmental coverage, it is unclear whether environmental effects and effects of stand structure on tree recruitment only apply to one forest type in one region or if observations are valid across regions with different climates or management regimes. Furthermore, climatic effects on tree recruitment have been addressed inconsistently across studies (Klopcic et al., 2012; Vayreda et al., 2013; Zell et al., 2019), which adds to the difficulties when comparing studies on tree recruitment.

Third, these studies usually focus on the abundant tree species and observations of less abundant species are aggregated based on the total abundance of several species (e.g., “other species” or “other deciduous”) instead of the ecological properties of a species (cf. Klopcic et al., 2012; Zell et al., 2019). This impedes systematic comparisons regarding successional strategies or species traits (e.g., shade or drought tolerance). For instance, studies on tree recruitment rarely focus explicitly on the relevance of shade tolerance (cf. Klopcic et al., 2014). There is little empirical evidence on how the tolerance to shade or drought modulates the response of tree recruitment to climate and stand structure at larger scales (Hanberry, 2019; Klopcic et al., 2014), although the importance of shade or drought tolerance for tree recruitment is beyond doubt from a qualitative and process‐based perspective (Leuschner & Ellenberg, 2017; Price et al., 2001; Shen & Nelson, 2018).

In order to address these limitations of previous studies on tree recruitment, we aim to analyze (a) how tree recruitment is related to stand structure and environment, and (b) how it changes with different levels of shade tolerance and drought tolerance across a wide range of environmental and forest stand conditions. We group species from an ecological point of view that allows for assessing the recruitment of all species in four data sets covering managed and unmanaged forests in Flanders (Belgium), northwestern Germany, and Switzerland. Furthermore, we consistently use one unified method across all data sets to study patterns of tree recruitment at different environmental ranges. This includes the verification of results from previous studies on tree recruitment across different regions and the quantification of qualitatively known patterns regarding successional strategies defined by the shade and drought tolerance of European temperate tree species. Specifically, we address three research questions.

1. What are the effects of basal area, stem density, and shade casting ability (SCA) of a forest stand as well as of degree‐day sum, water balance, and plant‐available soil water holding capacity on the recruitment rate? We expect that basal area, stem density, and shade casting ability determine the overall recruitment rate, while degree‐day sum, water balance, and plant‐available soil water holding capacity modulate the recruitment rate. Thus, effects of stand structure are expected to be stronger than climatic effects.
2. Do these effects act differently on groups of species with varying levels of shade and drought tolerance? Thus, effects of stand structure are expected to differ between shade‐tolerant and shade‐intolerant tree species. Specifically, increased basal area along with high stem density and high values of SCA should have strong negative effects in general with shade‐tolerant tree species less affected than light‐demanding species. Furthermore, we expect low water availability to have negative effects on tree species with low drought tolerance and less negative or positive effects for species with high drought tolerance.
3. What are the implications of using heterogeneous data sets for analyzing the abundance of recruitment in different regions and environmental settings? We expect different effects and levels of uncertainty between regions and data sets caused by the characteristics of each data set, for example, due to differences in forest types, environmental gradients, and sampling designs within each data set.

## 2 METHODS

### 2.1 Forest inventory data

**FIGURE 1:**
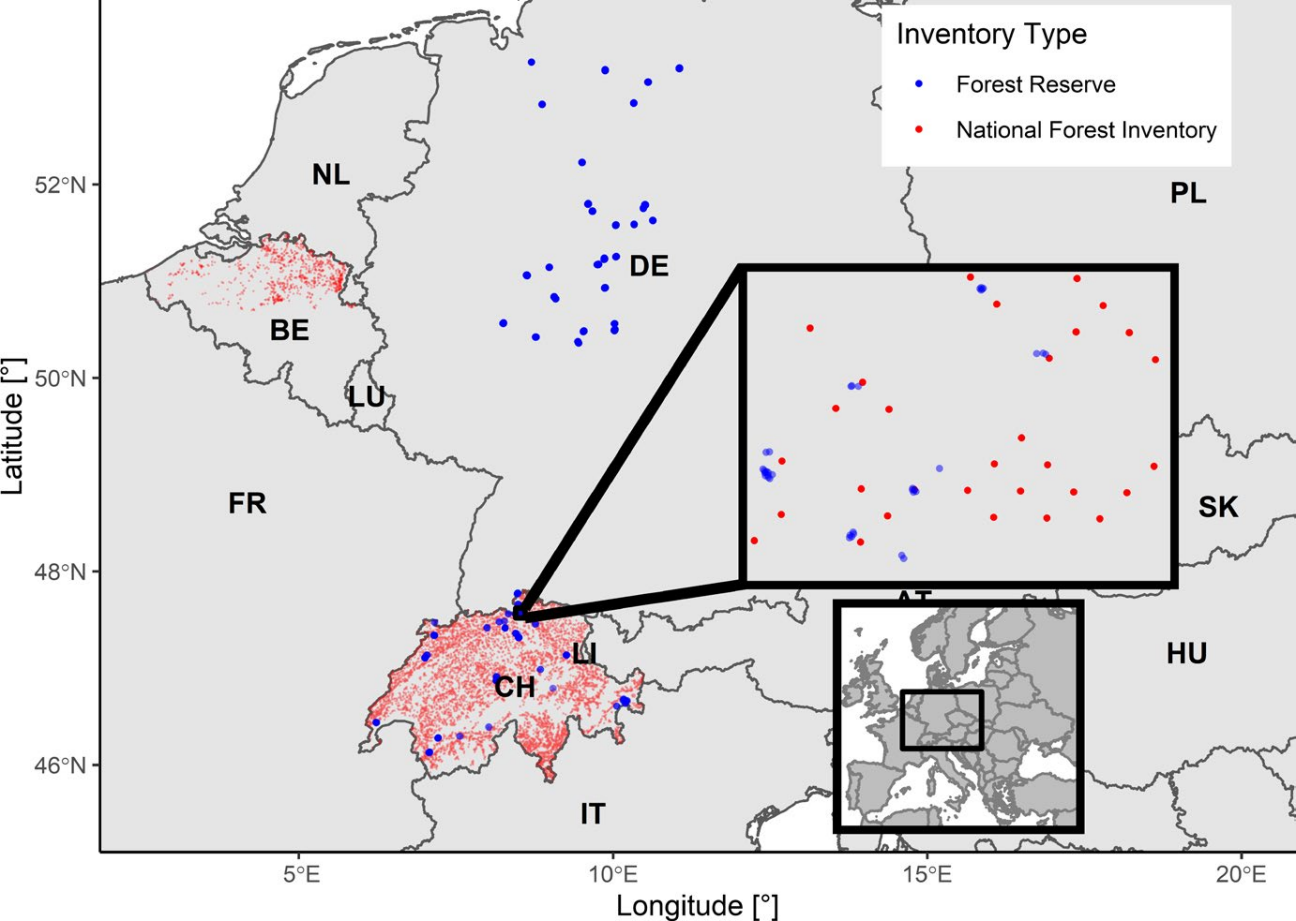
Map of the location of forest inventory plots projected with WGS 84 coordinates. Inventories are separated for National Forest Inventories (red) and Forest Reserves (blue). While for the National Forest Inventories all sample plots are shown in the large map, the locations of the Forest Reserves can contain multiple close plots (inset map)

Analyzing tree recruitment across different regions (Figure 1) and environmental scales requires a method to deal with heterogeneous data, which is most likely a key reason for the lack of large‐scale studies on tree recruitment based on multiple data sets. We selected four data sets that cover a wide range of forest types and inventory designs. These data sets specifically differ in sampling design, number of plots, plot size, the extent of the environmental gradient being covered, and the share of undisturbed natural forests (Tables 1 and 2).

**TABLE 1:** Properties of each data set

	CH FR	GER FR	FLAN NFI	CH NFI
DBH threshold for recruitment [cm]	4	7	7	12
Number of plots	258	1,320	837	4,519
Number of remeasurements per plot	1–6	1–2	1	1–3
Plots × remeasurements	574	1,529	837	9,939
Temporal range [calendar years]	1964–2018	1997–2018	2009–2019	1993–2013
Mean period length [years]	12.2	14.8	14.7	9.7
Plot size [m^2^]	277–34,704	500–1,000	200–254	200
Sampling design	Rectangular plots of varying size and position in different areas	Circular plots in different areas with regular grids of 100 × 100 m	Circular plots over the whole region on one regular grid 1.0 × 0.5 km	Circular plots over the whole region on one regular grid 1.4 × 1.4 km

Abbreviations: CH FR, Swiss forest reserves; CH NFI, Swiss national forest inventory; FLAN NFI, Flemish (northern Belgium) national forest inventory; GER FR, German forest reserves.

**TABLE 2:** Summary statistics of response variable “number of recruitment trees” and explanatory variables for all data sets

Variable	Unit	Data set	Percentile						
			0%	2.5%	25%	50%	75%	97.5%	100%
Number of recruitment trees per species and plot	1	CH FR	0	0	0	0	0	41	128
		GER FR	0	0	0	0	0	0	6
		FLAN NFI	0	0	0	0	0	3	31
		CH NFI	0	0	0	0	0	2	12
Plot size	ha	CH FR	0.03	0.05	0.23	0.35	0.55	1.26	3.47
		GER FR	0.05	0.05	0.10	0.10	0.10	0.10	0.10
		FLAN NFI	0.02	0.03	0.03	0.03	0.03	0.03	0.03
		CH NFI	0.02	0.02	0.02	0.02	0.02	0.02	0.02
Period length	years	CH FR	1	7	10	11	14	20	27
		GER FR	9	9	10	12	20	24	27
		FLAN NFI	10	10	13	14	17	20	22
		CH NFI	3	4	10	10	10	13	30
Basal area (≥DBH 4 cm)	m^2^/ha	CH FR	0.99	15.11	32.33	39.59	47.26	67.80	97.81
Basal area (≥DBH 7 cm)		GER FR	0.23	3.78	22.42	30.64	37.32	51.23	69.62
Basal area (≥DBH 7 cm)		FLAN NFI	0.40	1.65	11.76	21.33	30.93	49.22	72.07
Basal area (≥DBH 12 cm)		CH NFI	0.77	6.06	21.96	32.55	43.94	69.96	104.13
Stem density (≥DBH 4 cm)	1/ha	CH FR	140	253	586	863	1,258	2,404	3,146
Stem density (≥DBH 7 cm)		GER FR	10	50	140	240	410	1,080	2,480
Stem density (≥DBH 7 cm)		FLAN NFI	39	39	196	354	550	1,140	2,201
Stem density (≥DBH 12 cm)		CH NFI	20	60	220	340	510	960	2,310
Shade casting ability	[1–5]	CH FR	1.00	1.00	2.88	3.52	4.28	4.88	4.98
		GER FR	1.00	1.00	4.00	4.58	5.00	5.00	5.00
		FLAN NFI	1.00	1.00	1.00	1.81	3.00	5.00	5.00
		CH NFI	1.00	1.00	3.48	4.00	4.56	5.00	5.00
Mean of seasonal degree‐days (1971–2017)	°C·d	CH FR	966.29	1,099.03	2,131.37	2,800.73	2,965.20	3,130.25	3,130.25
		GER FR	1,933.54	1,947.98	2,484.01	2,619.90	2,755.72	2,908.35	3,053.68
		FLAN NFI	2,791.87	2,976.00	3,028.33	3,053.53	3,083.44	3,156.15	3,184.70
		CH NFI	583.63	1,184.45	1,984.14	2,428.20	2,785.48	3,108.91	4,362.37
Mean of seasonal water balance (1971–2017)	mm	CH FR	−378.74	−271.51	2.95	77.90	264.67	806.76	871.53
		GER FR	−250.59	−200.32	−120.37	−68.91	−4.59	286.20	311.20
		FLAN NFI	−216.54	−173.58	−155.26	−142.55	−130.82	−105.81	−42.29
		CH NFI	−365.09	−44.47	130.02	282.98	489.25	883.32	2,149.33
Bucket size	cm	CH FR	13.97	16.16	30.97	35.25	37.67	40.87	42.03
		GER FR	10.43	13.31	29.38	32.17	34.20	36.47	38.34
		FLAN NFI	9.34	11.28	14.81	18.17	24.91	36.15	44.19
		CH NFI	9.33	15.30	25.67	32.61	36.79	40.46	47.09
Elevation^a^	m a.s.l.	CH FR	336	346	499	625	1,105	1,983	2,104
		GER FR	13	20	233	411	535	872	904
		FLAN NFI	0	9	24	36	65	117	271
		CH NFI	194	428	647	954	1,322.5	1,950	2,220

Note

The number of recruitment trees was normalized by using plot size and period length in the models. Basal area and stem density are based on different DBH measurement thresholds; therefore, the threshold with which this variable was calculated is given in parenthesis (see Appendix S1: Section A2 and Figures A2 and A3 for a comparison with artificially increased DBH threshold). All variables were standardized and scaled for each data set to be comparable (Figure A1).

Abbreviations: CH FR, Swiss forest reserves; CH NFI, Swiss national forest inventory; FLAN NFI, Flemish (northern Belgium) national forest inventory; GER FR, German forest reserves.

^a^ Elevation was not used as explanatory variable and is shown to characterize the data sets only.

The tree recruitment rates and variables related to stand structure were derived from forest inventories in Flanders (northern Belgium), northwestern Germany, and Switzerland (Figure 1). The National Forest Inventories (NFIs) from Flanders (Wouters et al., 2008) and Switzerland (Abegg et al., 2014; Fischer & Traub, 2019) are subsequently referred to as “FLAN NFI” and “CH NFI,” respectively. Furthermore, two data sets from strict forest reserves (FRs, sensu Parviainen et al., 2000) in Germany (Meyer, 2005; Meyer et al., 2006, 2015) and Switzerland (Brang et al., 2011) are referred to as “GER FR” and “CH FR.” While the forest reserves consist entirely of unmanaged forests, the NFIs based on a gridded sampling plot inventory covering a very large region are dominated by managed forest but also consist of a representative fraction of undisturbed forests in Flanders and Switzerland (Sabatini et al., 2018). Specifically for the CH NFI, the fraction of unmanaged forests is 6% (Abegg et al., 2014; Portier et al., 2020). This is only mentioned here to raise awareness about the characteristics of the data sets. For the analysis, we used the whole NFI data sets including managed and unmanaged forests.

The two Swiss data sets cover the largest environmental gradient in our study due to the large elevational gradient in this country. Zell et al. (2019) present an analysis of tree recruitment with the Swiss NFI data. The Flemish (Belgian) data set mainly represents deciduous forests with similar climatic conditions between plots. The German data set complements the Flemish and Swiss forests with sandy soil conditions plus the sub‐montane forests of northwestern Germany.

### 2.2 Variable calculation

The annual recruitment rate was defined as the number of trees per ha and species that exceeded the calipering threshold (DBH threshold) for the first time between two consecutive inventories (Table 1). Tree species recruitment is not strongly correlated with nonrecruitment trees (i.e., local abundance, cf. Appendix S1: Figure A4 and Table A3). This suggests that the differentiation between tree recruitment and abundance of tree species is reasonable and the response variable can be considered a measure of recruitment and not species abundance.

Stand structure was characterized by the variables total basal area [m^2^/ha], stem density [ha^−1^], and shade casting ability (SCA) [value between 1 and 5] of the second census (i.e., the census in which recruits were observed for the first time). All trees that already had been defined as recruitment trees were not considered for the calculation of these variables. SCA is a species‐specific trait related to the ability of a species to cast shade, ranging from 1 (very low) to 5 (very high). It is based on factors such as leaf size, leaf angle, leaf density, and phenological aspects (see p. 185 in Leuschner & Ellenberg, 2017). The SCA values provided by Leuschner and Ellenberg (2017) were expanded to all species in our data set on a qualitative basis, considering the factors mentioned above. Overall for 15 out of 40 species, SCA values were not defined in Leuschner and Ellenberg (2017) and had to be complemented (see Appendix S1: Section A1, Table A1 for details). SCA at the plot level was defined as the mean of the SCA weighted by the share of each species with respect to stand basal area (cf. Depauw et al., 2021). Thus, SCA describes the effect of species composition on the light availability within a forest stand. The actual light availability depends on the variables basal area, stem density, and SCA. But none of these variables is an exclusive proxy for the available light and can only be interpreted together to infer on processes where light availability plays a role.

For the CH NFI and CH NFR inventory plots, environmental variables were calculated based on the swissALTI3D elevation data with a spatial resolution of 5 m (Federal Office of Topography, 2019) and interpolated climate data with a spatial resolution of approximately 1 km by MeteoSwiss (Federal Office of Meteorology and Climatology). Specifically, for precipitation the RhiresD data set (version 1.0) and for temperature the TabsD, TminD, and TmaxD data sets (all version 1.2) from MeteoSwiss were used. Climate data for the German and Flemish inventory plots originated from spatially downscaled gridded climate data with a resolution of 1 km based on the E‐OBS and WorldClim data sets (Moreno & Hasenauer, 2016). For the German and Flemish inventory plots, variables based on elevation data were taken from the EU‐DEM version 1.1 with a spatial resolution of 25 m (EU‐DEM, 2020).

Seasonal soil water balance was calculated according to the Thornthwaite method (Thornthwaite & Mather, 1957) for all months from April to October as the difference between monthly precipitation and monthly potential evapotranspiration (PET). Finally, the mean of the soil water balance from the years 1971 to 2017 was used as an explanatory variable. PET in the Thornthwaite model is based on the approach explained in detail by Bugmann (1994) and Fischlin et al. (1994) and was corrected for aspect and slope with

(1) $${\text{PET}}_{m,y}^{\prime }=kPMod\cdot {\text{PET}}_{m,y}$$

(2) $$kPMod=\left\{\begin{array}{ll} 1+kSlAsp\times 0.125, & kSlAsp>0\\ 1+kSlAsp\times 0.063, & \text{else} \end{array}\right.$$

where *kSlAsp* ranges from −2 to 2 and was calculated from a variable *s* based on the slope [°] with

(3) $$s=\text{MIN}\left(\frac{\text{slope}}{45}\times 2,2\right).$$

Subsequently, *s* was multiplied by −1 for north facing slopes and by 1 for south facing slopes in order to calculate *kSlAsp*.

The seasonal degree‐day sum refers to the annual sum of daily mean temperatures above 5.5°C for the months April to October, here averaged for the period 1971 to 2017. It was calculated for each plot with

(4) $$DDS_{y}=\sum_{d=\text{Apr}\;1\text{st}}^{\text{Oct}\;31\text{st}}\text{MAX}\left(T_{d,y}‐5.5,0\right),$$

(Allen, 1976). Calculations were performed for each year from 1971 to 2017. The average from all years was taken as a variable representing plot conditions.

Maximum soil water holding capacity (“bucket size”, BS) is based on soil depth, plant‐available water capacity, and coarse volumetric fraction. The calculation was adapted from Henne et al. (2011) and defined as:

(5) BS=SDR×AWC×(1‐CFV),

where SDR is soil depth to the bedrock for the R horizon with a maximum of 2 m, AWC refers to theoretical plant‐available soil water capacity based on the different soil compartments, and CFV is the volumetric fraction of coarse fragments. All soil information was derived from the ISRIC Soilgrid250 data set (Hengl et al., 2017). Qualitative evaluation of the bucket size revealed that absolute values were generally too high. Nevertheless, we decided to use the calculated bucket size because we assumed that the relative differences on larger scales are sufficient to represent a drought gradient.

### 2.3 Species groups

**FIGURE 2:**
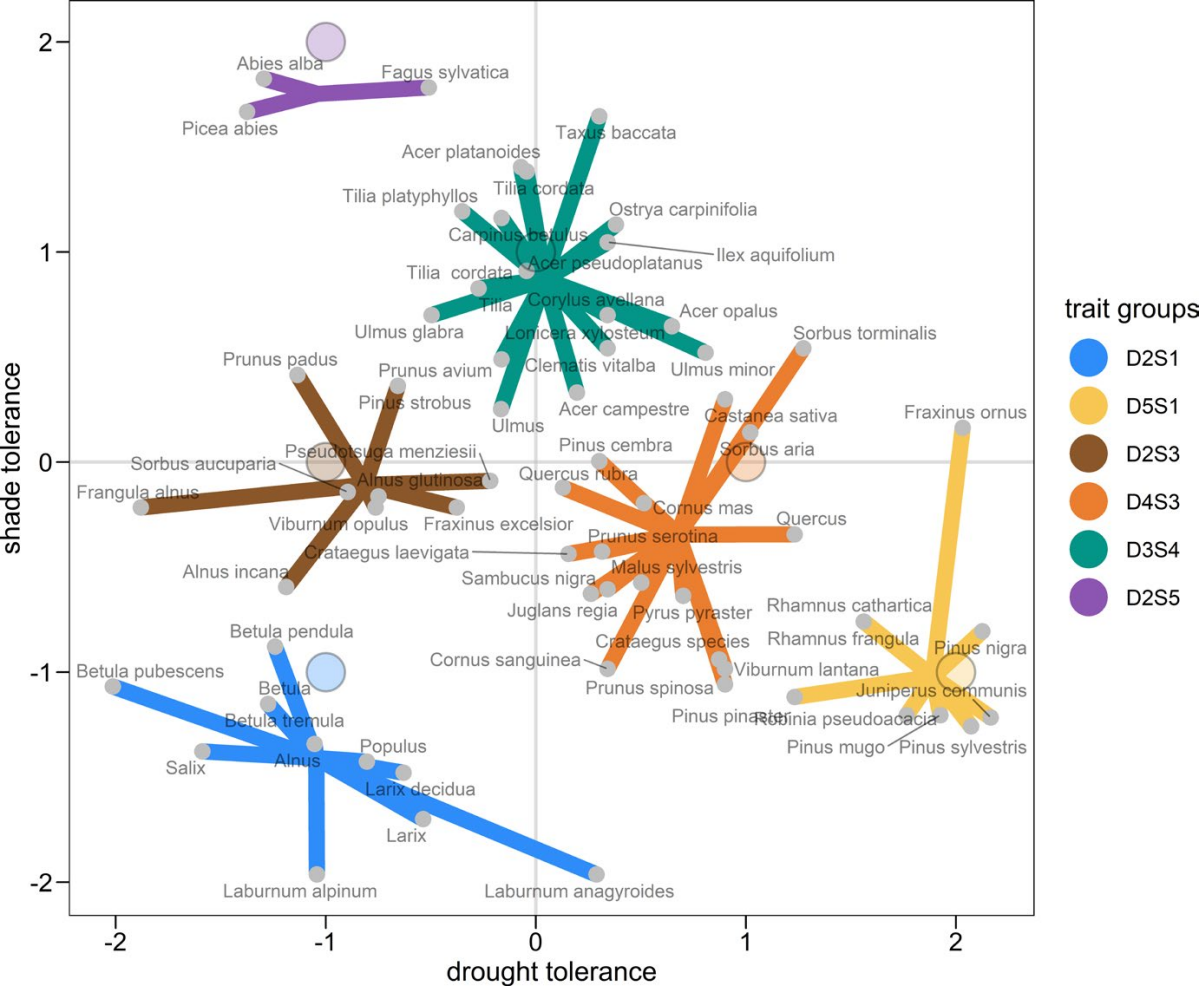
Grouping of species that occurred over all data sets using z‐scaled trait values. The large colored dots with black outline refer to the initial values used for the k‐mean clustering algorithm. Each trait group represents a group of species with similar tolerance to shade and drought. The contribution of different species within a trait group for the individual data sets is shown in Appendix S1: Figure A5 and Table A4

We defined six species groups according to their tolerance to shade and drought to differentiate between levels of shade tolerance and drought tolerance. These groups were subsequently treated as an attribute of a species, thus enabling the analysis of differences related to shade tolerance and drought tolerance without losing information (i.e., data) at the species level. The grouping was based on shade tolerance and drought tolerance values from the Functional Ecology of Trees (FET) data set (Niinemets & Valladares, 2006) and was derived from the TRY database (Kattge et al., 2020). We used shade tolerance values for juvenile trees, whereas the drought tolerance values were irrespective of the size or age of a tree species (Niinemets & Valladares, 2006). The definition of species groups was done in three steps. First, the trait values were standardized so that both values had a standard deviation of approximately two and an average value of zero across all species. Second, six initial points were defined in the two‐dimensional space spanned by the trait values of shade tolerance and drought tolerance (Figure 2). These initial values were defined by visual inspection of possible clusters and under consideration of ecologically plausible groups. This resulted in four trait groups on a diagonal from very shade‐tolerant to very drought‐tolerant and two trait groups for drought‐intolerant species with medium and low shade tolerance. Third, the species were grouped using the k‐means clustering algorithm (Hartigan & Wong, 1979). Detailed results on the final composition of trait groups are given in Appendix S1: Section A3, Figure A5, and Table A4. Some observations contained information at the genus level only. In these cases, the average trait values over all species within the FET database were used. For this reason, the trait values between genus and species might differ.

### 2.4 Modeling of tree recruitment

Our modeling procedure was applied to all data sets separately and resulted in four separate model fits. In addition, we fitted three models based on an artificially increased DBH threshold (12 cm) for CH FR, GER FR, and FLAN NFI to assess the implications of using different DBH thresholds between models. All variables except plot size and period length were transformed to be on the same scale and make the coefficients comparable (see Appendix S1: Section A2; Figure A1). The response variable “number of recruited trees” contains many incidences without recruitment (number of recruited trees = 0) that is, the data are zero‐inflated. Zero‐inflation can violate model assumptions if the probability distribution does not allow for frequent zeros. Most previous studies dealt with zero‐inflation using a two‐stage modeling approach, that is, a hurdle model where the zero‐producing process (the occurrence of recruitment) and the count process (number of recruited trees) are separated (Klopcic et al., 2012; Mugasha et al., 2017; Vanclay, 1992; Yang & Huang, 2015). This is achieved by modeling a binary process and using zero‐truncated probability distributions. Others argued that zeroes originate from both processes and hence cannot be separated entirely (Fortin & DeBlois, 2007; Zell et al., 2019; Zhang et al., 2012). They also advocated the use of a binary and a count probability distribution but allowed for zeroes in the probability distribution for the counts.

We evaluated several probability distributions such as negative binomial and Poisson with and without zero‐inflation or as hurdle models. All distributions allow for a large number of zeroes but based on the assessment of scaled residuals (Hartig, 2020) the negative binomial distribution proved to be the best choice. Specifically, we visually inspected the expected values versus the observed values and chose the distribution with the least systematic deviations (Appendix S2: Figure B1). Nevertheless, although the negative binomial distribution still did not fit the data perfectly for the largest response values, it constituted the best compromise for identifying one probability distribution suitable for all data sets.

We aimed to include the most important environmental factors for modeling tree recruitment and considered different variables such as slope, aspect, temperature, radiation, precipitation, plant‐available water capacity, soil compartments, and soil depth. However, we decided against a systematic model selection to facilitate the comparisons among the four models. Instead, we evaluated the ecological relevance, interpretability, and the ecological relationship between all variables. As a result, slope, aspect, precipitation, and temperature were combined to calculate the seasonal water balance. The soil‐related variables were summarized in the “bucket size” variable. Subsequently, we assessed the collinearity between these variables by calculating Variance Inflation Factors (VIF, Naimi et al., 2014), resulting in the final set of variables for which the VIFs were below the critical threshold of 10 (cf. Dormann et al., 2013 and Appendix S1: Table A2). Only the variable water balance was removed from the GER FR data set because of the high correlation with the degree‐day sum (cf. Table A2). Otherwise, we applied the same model structure with the same variables to all four data sets.

Two interactions were included for variables where the interpretation of the ecological relationship is straightforward. Basal area and stem density interact by describing the actual available space in a forest stand. High basal area and low stem density describe stands with some very large trees (mostly older trees) but high basal with high stem density describes stands with many smaller trees (mostly younger trees). It is important that the model can differentiate between such cases. Water balance and bucket size interact by determining the actual available water at a site. If one of these variables is low, the other variable may compensate and contributes to a higher level of water availability at a site. We did not include further interactions among stand structure or climate although they might lead to significant results because these interactions involve various processes and feedbacks that impede inferring on causal relationships.

All observations were treated at the species level to account for species‐specific differences inside trait groups, but trait groups were used as grouping variable for the statistical analysis. For each plot observation, plot size [ha] and the period length defined by the number of years between two consecutive inventories were used to account for systematic offsets in the models.

The model was defined as

(6) $$Y_{i,t,j}∼\text{NB}\left(\mu_{i,t,j},\phi \right),$$

where we assumed that the observed number of recruitment trees *Y* per plot *i*, year *t,* and species *j* followed a negative binomial distribution (NB) defined and parameterized by the mean *μ* and dispersion parameter *ϕ* according to chapter 14.2. in the function references of the Stan documentation (Stan Development Team, 2018).

The expected number of recruits was modeled as:

(7) $$\log\left(\mu_{i,t,j}\right)=\log\left(A_{i,t}\times \Delta t_{i,t}\right)+\beta_{0,g}+u_{0,i}+u_{0,j}+\sum_{m=1}^{N_{\text{vars}}}\left(\beta_{g}^{\left(m\right)}+u_{j}^{\left(m\right)}\right)x_{i,t}^{\left(m\right)}$$

$\text{log}\left(A_{i,t}\times \Delta t_{i,t}\right)$ refers to the model offsets that are based on plot size $A_{i,t}$ and period length $\Delta t_{i,t}$ and $\beta_{g}^{\left(m\right)}$ are the coefficients for each trait group *g* and variable *m* where $N_{\text{vars}}$ is the number of variables. $x_{i,t}^{\left(m\right)}$ is the observed value of the explanatory variables *m*, where the stand structure‐related variables (i.e., basal area, stem density, and SCA) vary between each plot *i* and year *t*. The environmental variables degree‐day sum, water balance, and bucket size varied between plots *i* only. Random intercepts $u_{0Ii,j}$ were added for each ploI *i* and species *j* and random slopes $u_{j}^{\left(m\right)}$ for each species *j* and variable *m* to account for species‐specific differences within trait groups. All random effects were assumed to be normally distributed:

(8) $$u∼N(0,\sigma^{2}).$$

Due to the complex random effects, structure, and the large number of model coefficients the models did not converge using maximum likelihood as implemented in the glmmTMB package (Brooks et al., 2017). Thus, the models were implemented using the *brms* package (Bürkner, 2017, 2018; Stan Development Team, 2018) with the statistical software *R* (version 4.0.3, R Core Team, 2020). An example of the code used to specify the model is given in Appendix S2: Section B1. Coefficients were estimated based on maximum likelihood using a Monte Carlo Markov Chain (MCMC) sampling algorithm with four chains, each with 5,000 iterations including a burn‐in phase of 2000 iterations. Detailed information on the results of the sampling process is provided in Appendices [Link], [Link]. We assessed model performance by comparing the differences between 1,000 model simulations and observations and evaluating both the number of zeroes and the distribution of the simulated counts. The duration for fitting the models was between one and ten days, which impeded further model evaluation, that is, calculating *R* ^2^ or conducting a cross‐validation. Since the objective of this study was inference and not prediction, this constraint is acceptable. To reduce autocorrelation of the MCMC samples, we only used 3,000 of the 12,000 MCMC samples after the burn‐in phase to assess the fit of all models (cf. Appendix S2: Figure B2). Specifically, we calculated credible intervals, the mean as the central tendency (i.e., effect estimate) and simulated the number of recruitment trees for each variable and trait group with the remaining variables set to their mean. For these simulations, we considered values within the range of the 1st to 99th percentile of observations of the explanatory variables per trait group (including nonrecruitment trees). The mean and the credible intervals were used to assess the response of trait groups to the explanatory variables.

## 3 RESULTS

All models converged with the Gelman‐Rubin diagnostic being below the critical threshold of 1.01 for all parameters (Gelman & Rubin, 1992), which indicates that all MCMC chains cover the same parameter range (i.e., the stationary distribution) and that the variation between chains is similar to the variation within chains. In combination with the visual inspection of the MCMC sampling trace plots, this indicates good mixing of the parameter samples. Inspection of the residual plots (Appendix S2: Figure B1) revealed that the models generally overestimated high recruitment rates. Furthermore, plot‐level random effects caused patterns in the residuals. Based on the detailed inspection of these patterns in the CH FR data, we found that a quadratic effect for shade casting ability (SCA) was missing for trait groups D3S4 and D2S5. This implies that the recruitment rate of very shade‐tolerant tree species was positively affected by very low and very high values of SCA. However, after testing model fits including quadratic effects for SCA, these patterns did not disappear as the quadratic effect did not apply to all trait groups and was apparent in the CH FR data set only. We therefore decided not to account for this effect within the model and to accept these minor inconsistencies in favor of consistent models across the four data sets. Fortunately, the comparison between nonzero observed tree recruitment rates and simulated tree recruitment rates across trait groups and data sets showed that the deviations of the fitted models from the observations were acceptable (Figure B1). Also, the number of simulated zeroes was close to the expected numbers.

### 3.1 Effects of stand structure and climate on tree recruitment

**FIGURE 3:**
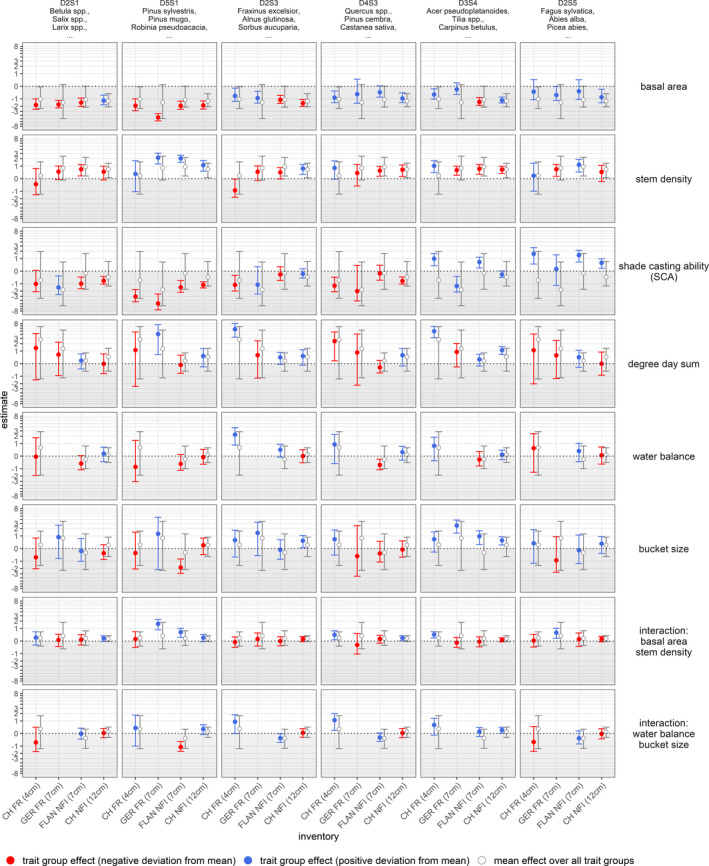
Effect estimates drawn from 3,000 posterior samples. Expected mean effect size across all trait groups (open circles) and trait group‐specific deviation (full circles) with 95% credible intervals (whiskers) are shown. Trait group‐specific mean estimates that were larger than the mean effect over all trait groups are in blue, whereas negative deviations are in red. For better understanding, the most abundant species for each trait group are mentioned after the trait group acronym at the top panels, but all species within one trait group were considered in the models (see Figures 2 and A5 or Table A4). For species‐specific random effects, see Appendix S3. CH FR, Swiss forest reserves; CH NFI, Swiss national forest inventory; FLAN NFI, Flemish (northern Belgium); GER FR, German forest reserves. Note that tree recruitment, basal area, and stem density were based on different DBH thresholds for each data set: CH FR ≥ 4 cm, GER FR ≥ 7 cm, FLAN NFI ≥ 7 cm, and CH NFI ≥ 12 cm. The vertical axis was transformed using the log‐modulus transformation (John & Draper, 1980) to facilitate comparisons between trait groups

**FIGURE 4:**
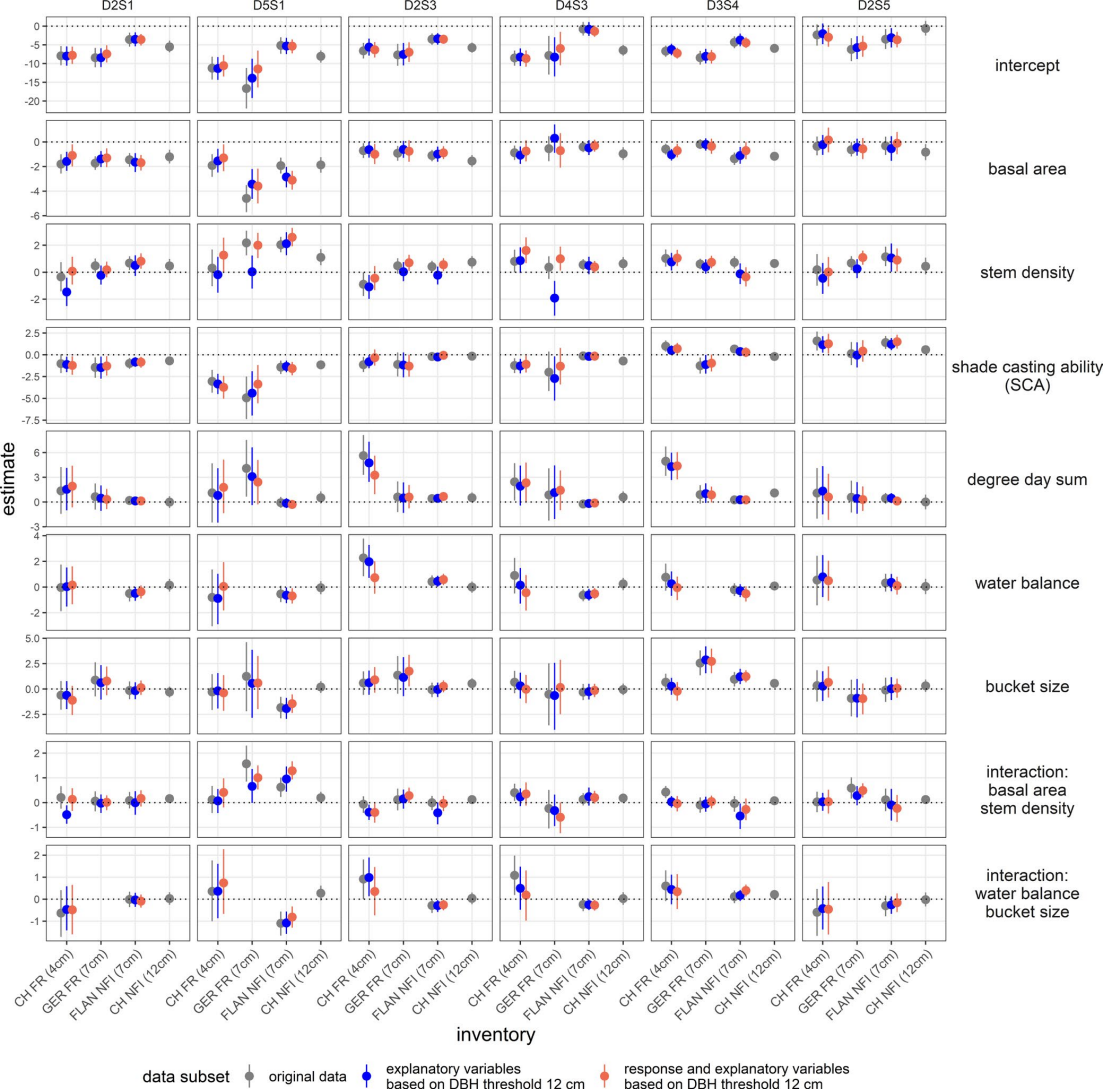
Comparison of models with modified inventory data. Shown are expected effect sizes (dots) with 95% credible intervals (vertical lines). Model fits based on the original data sets without modified DBH are shown in gray. Model fits based on data where the DBH threshold for explanatory variables was artificially increased to 12 cm are shown in blue. Model fits based on data sets where both explanatory variables and abundance of tree recruitment were calculated with a DBH threshold of 12 cm are shown in red. Note that the gray bars correspond to the same values shown in the trait group‐specific values shown in Figure 3. For a description of the trait groups (D2S1, D5S1, D2S3, D4S3, D3S4, D2S5) see Figure 2, Table A4, and Figure A5

**FIGURE 5:**
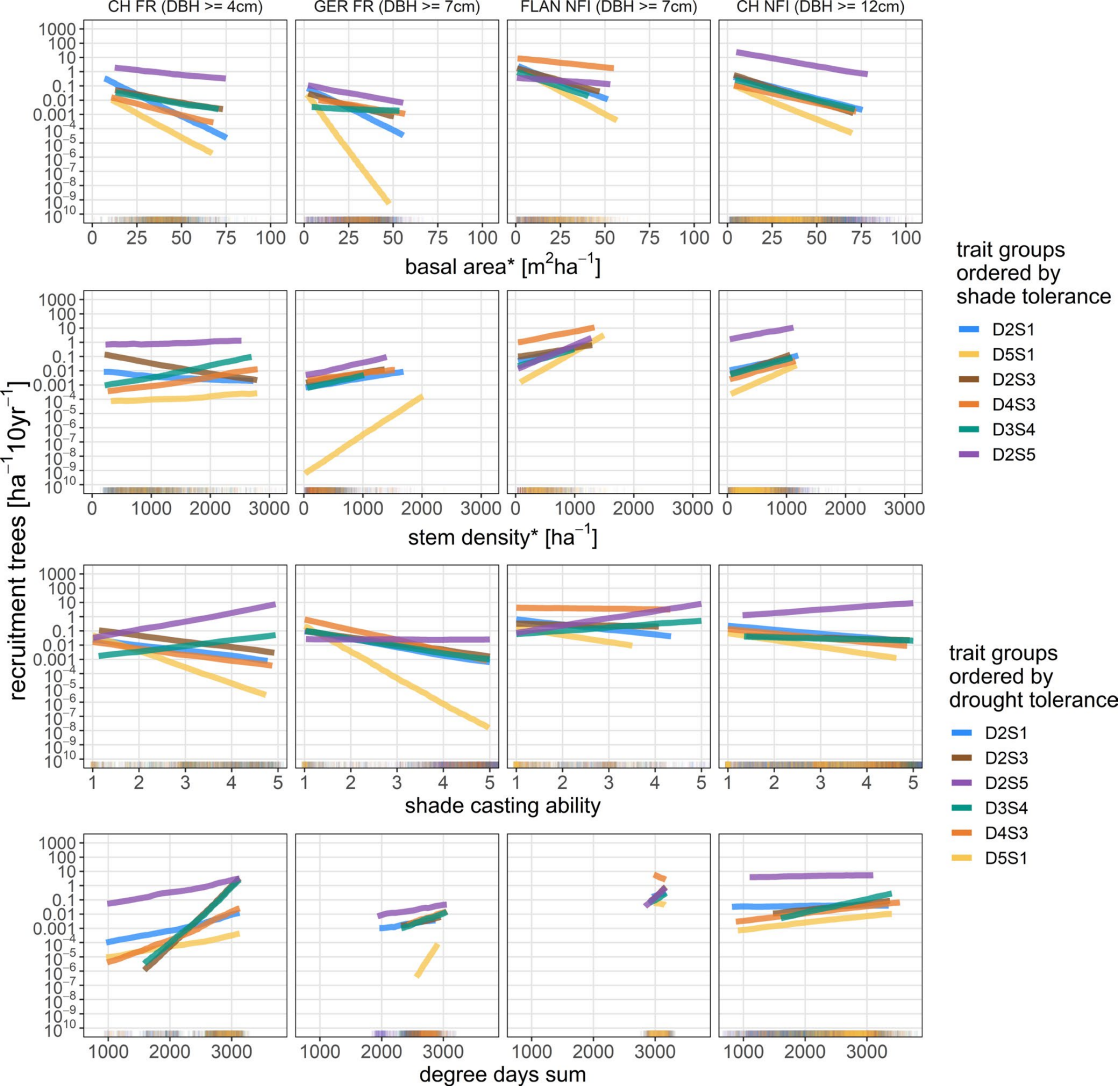
Simulations for each variable with all other variables set to their respective mean. Only values within the range of the 1st to 99th percentile for each trait group were considered. Rugs at the bottom axis indicate observations where there is at least one item (including nonrecruitment) of a given trait group. We added noise of 5% to the rugs to avoid overlapping of trait groups. Note that we chose to exclude water balance and bucket size from this figure due to the large credible intervals associated with these variables (see Figure 3). *Tree recruitment, basal area, and stem density were based on different DBH thresholds for each data set. These are given in the title above the top panels. The simulation results showing credible intervals and results for variables not included in this figure can be found in Appendix S2: Section B4; Figures B3–B9. For a description of the trait groups (D2S1, D5S1, D2S3, D4S3, D3S4, D2S5) see Figure 2, Table A4, and Figure A5

**FIGURE 6:**
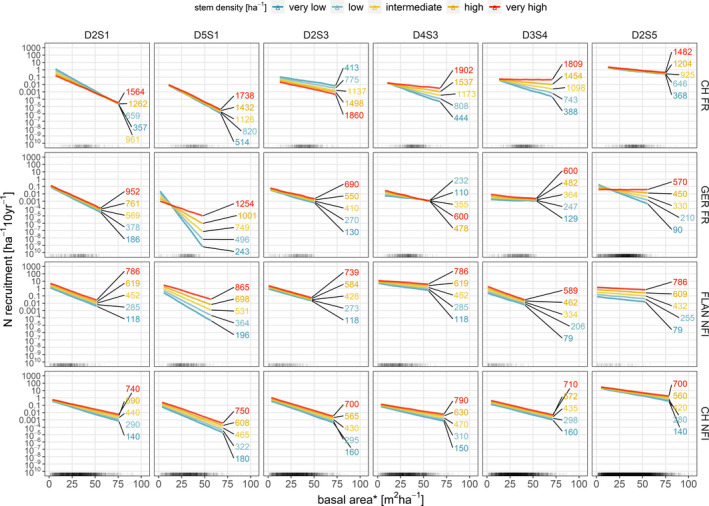
Simulated interaction between basal area and stem density. Basal area values range from the 1st to the 99th percentile of values where a trait group was observed (both recruitment and nonrecruitment). Stem density is shown for the 10th (very low), 30th (low), 50th (intermediate), 70th (high), and 90th (very high) percentile. CH FR, Swiss forest reserves; CH NFI, Swiss national forest inventory; FLAN NFI, Flemish (northern Belgium) national forest inventory; GER FR, German forest reserves. *Note that Tree recruitment, basal area, and stem density were based on different DBH thresholds for each data set: CH FR ≥ 4 cm DBH, GER FR ≥ 7 cm DBH, FLAN NFI ≥ 7 cm DBH and CH NFI ≥ 12 cm DBH. Rugs at the bottom axis indicate observations where at least one individual (including nonrecruitment) of a certain trait group exists. We added noise of 5% to the rugs to avoid overlapping of trait groups. Figure B9 shows a similar graph for water balance and bucket size but is not included in the main text due to the large credible intervals. For a description of the trait groups (D2S1, D5S1, D2S3, D4S3, D3S4, D2S5) see Figure 2, Table A4, and Figure A5

Tree recruitment was more affected by basal area, stem density, and SCA than by climate, with the latter being subject to substantial uncertainty (Figure 3 and Table B1). Furthermore, differences between trait groups were mostly associated with different levels of shade tolerance. However, our results indicated no general differences between levels of drought tolerance. The models based on harmonized DBH thresholds resulted, with few exceptions for basal area and stem density, in minor differences only, thus suggesting that comparisons among the models are valid. Furthermore, this indicates that the same factors at the same magnitude govern recruitment at DBH thresholds of 4, 7 or 12 cm (Figure 4). Effects related to stand structure were consistent across data sets, but climatic effects varied between data sets with a considerable degree of uncertainty (Figures 3, B6 and B7). The differences between trait groups and the similarities between data sets in terms of uncertainty were surprisingly consistent in this respect. The effects of stand structure across trait groups were similar for all data sets (Figures 3, 5 and 6). However, differences emerged when the effects for trait groups associated with different levels of shade tolerance were compared (Figures 3 and 5), as explained below.

Estimates for basal area were negative, but distinctly stronger for the light‐demanding species of trait groups D2S1 and D5S1, with the exception for trait group D2S1 in the CH NFI data set. In addition, positive deviations from the mean were apparent for shade‐tolerant species of trait groups D3S4 and D2S5, again with an exception, this time for trait group D3S4. Stem density was positively associated with the recruitment rate for all trait groups in all data sets with almost no differences between trait groups, with the exception of the estimates for trait group D2S3 from the CH FR data set, which were negative (Figures 3 and 5). The most likely reason for this exception was one observation with an exceptionally high abundance of *Fraxinus excelsior* recruitment in combination with the low DBH threshold of CH FR (cf. Appendix S2: Figure B8 and random effects within this trait group shown in Appendix S3: Figure C3). The interaction between basal area and stem density was on average and over all trait groups positive, implying that the negative effect of basal area was stronger for low values of stem density (Figure 6). Apart from the outlier caused by *F. excelsior* in trait group D2S3 in CH FR (cf. above), there were no deviations from the average interaction effect between trait groups.

The recruitment response to shade casting was generally negative, but with strong deviations from the mean over all trait groups, especially for light‐demanding and shade‐tolerant trait groups (Figures 3 and 5). While the negative effects of shade casting were stronger for the light‐demanding species of trait groups D2S1 and D5S1, the shade‐tolerant species of the trait groups D3S4 and D2S5 in contrast responded positively to high values of the SCA. These deviations were apparent in all inventories and strongest for trait groups D5S1 and D2S5, whereas the moderately shade‐tolerant species of the trait groups D2S3 and D4S3 did not substantially differ from the average negative response to shade casting.

In contrast, the effects of environmental variables on tree recruitment were less clear and the associated level of uncertainty was considerably high, indicated by the large credible intervals or the inconsistency in effect sizes across data sets (panels of rows 4, 5, and 6 in Figure 3 or Figures B6–B8). Higher degree‐day sum was on average and over all trait groups positively associated with tree recruitment across all data sets (Figures 3 and 5): Clear deviations from the mean were only apparent within the CH FR and the CH NFI data for trait group D3S4 (5th column 3rd row Figures 3 and 5) and D2S3 within the CH FR (3rd column 3rd row in Figures 3 and 5). Effects of water‐related variables differed between data sets, trait groups, and variables (Figure 3). Although the mean estimates for bucket size tended to be positive, describing these effects in terms of water availability became challenging due to the interaction with water balance (Figures 3 and B9) in combination with the large credible intervals (Figures 3, B6–B8). Only for some trait groups in the GER FR and FLAN NFI data set, there were positive relationships with bucket size (Figures 3 and B8). However, given the missing evidence for these effects in the other data sets and the lack of consistency across trait groups, the main finding for the variables water balance and bucket size was that hardly any unifying effects on tree recruitment could be identified.

## 4 DISCUSSION

Basal area, stem density, shade casting ability (SCA), and the degree‐day sum influence tree recruitment rates similarly across the four study regions and along wide environmental gradients. Moreover, pooling multiple species based on their shade tolerance revealed consistent patterns of the relationship between recruitment and stand structure. Specifically, basal area along with species’ shade casting ability proved to be important stand characteristics for predicting differences in recruitment between successional strategies expressed by shade tolerance. A higher degree‐day sum was related to higher recruitment rates when assessing all trait groups, but regarding drought tolerance no evidence for differences between trait groups was found in our study. Aspects related to the water balance of a site turned out to be subject to large uncertainty. Overall, our results show that tree recruitment is primarily driven by stand attributes such as basal area and stem density as well as by the shade casting ability of the constituent species in the overstory. This was evident in all four data sets in spite of the large heterogeneity resulting from varying assessment methods and environmental ranges.

Recruitment of trees into size classes of ≥4 cm DBH, as in our four data sets, represents a relatively late stage of forest regeneration. It integrates over sometimes long periods since germination and multiple environmental constraints act upon young trees of widely different size. This causes the statistical relationships between stand structure, climate, and tree recruitment to be manifold and complex. In the following, we discuss whether our models allow to infer on causal relationships for each variable and trait group and how underlying ecological processes explain the estimated effects.

In addition, we compare our results to other studies. One of these studies is the one from Zell et al. (2019) which we briefly summarize here because it also analyzed the NFI data with a different analytical approach. Specifically, they modeled the number of recruits independent of the species composition of recruits. Zell et al. (2019) also included climate in their analysis and found that temperature and precipitation had minor negative effects on the number of recruits, whereas the temperature had strong effects on tree recruitment species composition. Furthermore, they identified that the most dominant species is a very important explanatory variable for the species composition. The effects regarding basal area and stem density reported by Zell et al. (2019) are generally confirmed by our findings. More details on the comparison with the study from Zell et al. (2019) and other studies are provided throughout the discussion.

### 4.1 Effects of stand structure on tree recruitment

Our findings regarding the relationship between basal area and tree recruitment are consistent with previous studies (Klopcic et al., 2012; Vanclay, 1992; Zell et al., 2019). Several aspects related to competition for resources below‐ and aboveground may cause a negative response of tree recruitment rates to increased stand basal area (Casper & Jackson, 1997; Mina et al., 2018) with light availability being particularly important in this respect (Adams et al., 2007; Monsi, 2004). Altogether, basal area serves as a proxy for the competitive situation in a forest stand resulting from its development stage, that is, older stands with large trees tend to have higher values of basal area (cf. Feldmann et al., 2018; Glatthorn et al., 2018; Pretzsch, 2009). Nevertheless, basal area cannot be interpreted without considering SCA and the interaction with stem density, because basal area alone is not a perfect proxy of competition as it integrates over a wide range of forest stand properties. The consistent negative effect of basal area, however, underlines the paramount importance of this variable for understanding forest regeneration dynamics (Klopcic et al., 2012; Vanclay, 1992; Zell et al., 2019).

The estimated effects for SCA are, together with basal area, most consistent for all models and trait groups. Like basal area, the effect estimates reflect a gradient of shade tolerance among trait groups. Differences between trait groups are also considerably larger than those for basal area, which is what we expected when considering the ecological processes determining these variables. In contrast to basal area, the SCA of a species is related to the intensity of competition experienced by tree recruitment for the resource light. It is indicative of the relative amount of light that penetrates the canopy, given a certain value of basal area, and is thus strongly modulated by species composition (Leuschner & Ellenberg, 2017). Apart from that, the SCA is also related to the abundance of conspecific trees or at least trees of similar successional strategies in terms of shade tolerance (cf. Table A1). To understand the effect of this variable, one therefore needs to consider its implications in extreme situations. For example, if a forest consisted of *Betula pendula* only, this would result in the lowest possible value of SCA, whereas actual light availability at the forest floor is co‐determined by basal area and stem density. This is the reason for the residual patterns indicating a nonlinear effect of SCA in the CH FR data (cf. Figure B6).

The estimated effects of the SCA confirm that, in the absence of large disturbances, stands develop toward a state where shade‐tolerant species are more dominant, that is, the shifting mosaic steady‐state theory (SMSS, Bormann & Likens, 1979; Shugart & West, 1981). Furthermore, the concept of different life strategies such as competitor, stress tolerator, and ruderal (CSR, Grime, 2006; Halpern, 1989; West et al., 1981) is supported by our finding that distinct differences in effects of SCA between levels of shade tolerance exist. This is specifically evident from the finding that larger SCA (i.e., the dominance of late successional tree species) has negative effects on the recruitment of early successional tree species but positive effects for late successional tree species (see Figure 5). This contrasting pattern underpins the importance of shade casting for the recruitment of trees into larger size classes (DBH ≥4 cm). Our findings complement the results of previous studies dealing with the effect of shade casting on the forest herb layer (e.g., Verheyen et al., 2012) and on tree seedlings (e.g., De Lombaerde et al., 2019). While the relevance of SCA is plausible from an ecological perspective, it is represented unexpectedly well in all study regions regardless of the assessment method, the environmental conditions, and the observed species composition.

Stem density is an important variable to describe stand development stage, jointly with basal area (Pretzsch, 2009). While basal area is generally more informative regarding the development stage of a forest, stem density alone is a problematic variable when analyzing the tree recruitment rates because distinguishing between tree recruitment being a consequence or a cause of high stem density is difficult. For this reason, stem density can only be interpreted by considering its interaction with basal area. We found that stem density generally has a positive effect on recruitment of trees for the size classes considered here (DBH ≥ 4 cm) but this effect is becoming weaker with increasing basal area (Figure 6). This is consistent with the findings by Zell et al. (2019), but comparisons to other studies are difficult because tree density is not usually used for modeling tree recruitment. Instead, mean tree diameter is often used (Klopcic et al., 2012, 2014; Yang & Huang, 2015), which is comparable with stem density only under the assumption that all stands are even‐aged, which is clearly not the case in our data sets. Additionally, mean tree diameter is usually associated with basal area and therefore its effects are difficult to distinguish from those of stem density.

Low stem density should result in large amounts of available light and space for recruitment. However, in our study stem density was, with the exception of trait group D2S3 in the CH FR data, positively associated with recruitment. There are two possible albeit interdependent explanations for the effects of stem density. First, tree recruitment can take place in a time span that exceeds the inventory periods, such that a high abundance of tree recruitment in earlier inventories resulted in a high stem density while tree recruitment is still going on. Second, the interaction between stem density and basal area modulates the effect of stem density and is best explained by considering two extreme cases. A forest stand can have low stem density for two reasons: (a) a disturbance caused gaps in the stand and recruitment did not yet pass the diameter threshold to fill the gaps or (b) a few very large trees suppress the emergence of other trees. All stages between these two cases can be considered forest development stages as a function of stem density and basal area. Feldmann et al. (2018) and Glatthorn et al. (2018) support this explanation by showing that stem density is lowest for later stages of forest development. The relevance of the interaction between basal area and stem density for modeling tree recruitment was also demonstrated by Zell et al. (2019). Considering both explanations, the relationship between stem density and tree recruitment is mostly driven by aspects related to stand level dynamics and not species attributes, which is also supported in our models by the absence of trait group‐specific differences in effect size for stem density. Another possible explanation is that along elevational gradients trees get smaller‐statured which results in higher stem density and higher mortality. Thus, turnover rate including tree recruitment increases. This explanation however is not supported by the overall positive effect of degree‐day sum.

### 4.2 Climatic effects on tree recruitment

We explain the positive effect of degree‐day sum by an increased turnover rate. If no other biotic or abiotic factors are limiting, all tree species profit from higher temperature because of higher photosynthesis rates that lead to increased growth rates. Nevertheless, the effect of degree‐day is difficult to link to specific processes without considering water‐related variables. Although we demonstrated a generally positive effect of the degree‐day sum on tree recruitment, which confirms the findings in Zell et al. (2019), the evidence for distinct effects of water relations on tree recruitment across large environmental gradients was rather elusive. Also, no differences in the effect size for the degree‐day sum were identified between levels of drought tolerance, despite the fact that temperature is an important driver of drought stress (Martin‐Benito & Pederson, 2015; Williams et al., 2013). The difficulty in finding consistent effects of water relations on tree recruitment is underpinned by the fact that most empirical evidence for such effects are based on regional studies (Ibáñez et al., 2007; Kroiss & HilleRisLambers, 2015; van Mantgem et al., 2006). Besides Zell et al. (2019), there are no larger‐scaled studies that considered other variables than precipitation for representing water relations when modeling tree recruitment. However, our findings confirm Zell et al. (2019), which also found that water holding capacity was not useful for modeling species composition of tree recruitment. Furthermore, the complexity of climatic effects on tree recruitment is evident from various studies. Germination and growth is mostly positively affected by temperature (Hobbie & Chapin, 1998; Munier et al., 2010; Zurbriggen et al., 2013) but effects of temperature on survival can be positive, negative, or neutral (Loranger et al., 2016; Munier et al., 2010; Zurbriggen et al., 2013). Effects of water availability are even more complex and show contrasting patterns for germination, growth and survival of seedlings (Lett & Dorrepaal, 2018). Apart from the lack of consistent findings from our as well as previous studies, it is also questionable if the drought gradient covered in our study was sufficient to find a clear signal on water relations.

What is the reason for climatic effects being so poorly represented in our study? One possible explanation is that trees of a species may experience ontogenetic shifts, that is, they are able to germinate and grow under certain climatic conditions but do not necessarily survive under the same climatic conditions until they reach a size class that is considered tree recruitment (cf. Schupp, 1995). For example, Máliš et al. (2016) showed that the environmental range between seedlings and saplings or older trees differs. Another explanation is that microclimate is more relevant for successful recruitment than climate outside the forest stand (von Arx et al., 2013; De Frenne et al., 2019; Zellweger et al., 2020). This would also explain the strong effect of SCA that directly affects microclimatic conditions on the forest floor. Accounting for the uncertain effect estimates, the ontogenetic differences to deal with climatic stress and the relevance of microclimate, a nonlinear, complex relationship between climate and tree recruitment appears to be the most adequate explanation for our results.

Besides the individual effects of each variable, there is also variation of species‐specific effects within each trait group (Appendix S3). Although our grouping regarding shade tolerance yielded plausible results, the groups based on drought tolerance did not allow for valuable conclusions. However, although our study did not reveal simple linear relationships or general patterns between climatic variables and trait groups, we showed that the effects of water relations on tree regeneration are manifold and may depend on species‐specific properties that may not have been evident in the definition of our trait groups or may vary in a nonsystematic fashion with site properties.

### 4.3 Potential effects of soil fertility and forest management

Other aspects that were not dealt with explicitly in our analysis are soil fertility and the effect of forest management. Soil fertility was not addressed because the focus of this study was to identify the importance of tree species tolerance to shade and drought for tree recruitment. For this reason, variables directly related to shading or drought were preferred. However, we acknowledge that increased soil fertility may increase seedling survival in the shade for some tree species (cf. Kobe et al., 1995; Walters & Reich, 2000) and therefore increase the partly positive effects of SCA for very shade‐tolerant tree species or weaken the negative effect of basal area. Initially, the effect of forest management was considered to be addressed explicitly in our analysis because the two data sets from FRs and two data sets from NFIs differ fundamentally in this respect. However, the heterogeneity of the data sets and the very different sampling designs between FRs and NFIs impeded general comparisons between managed and unmanaged forests. Furthermore, we decided against conducting a joint analysis with FR (unmanaged) versus NFI (mainly managed) as a variable because possible effects between inventory designs would have been difficult to disentangle from the effects of management. Additional difficulties for such an analysis are that NFIs also cover unmanaged forests (cf. methods) and that harmonizing the DBH threshold would result in a considerable loss of information. In our analysis, the only clear difference between FRs and NFIs was that the negative effect of basal area on tree recruitment for trait groups D2S3 and D3S4 was stronger in NFIs (managed forests). This indicates that species of these groups such as *Fraxinus excelsior*, *Alnus glutinosa*, *Acer pseudoplatanus*, or *Tilia cordata* have a reduced abundance in favor of other species, due to forest management practices that also include tending measures in regeneration.

### 4.4 The relevance of diameter measurement thresholds for records of tree recruitment

We expected substantial differences between results of the different data sets because diameter measurement thresholds were not equal. However, this was not the case as the estimated effects did not vary substantially between data sets with diameter measurement thresholds of 4, 7, and 12 cm (Figure 4). This finding is contrary to a study on biomass recruitment where a systematic bias caused by different size thresholds was found (Searle & Chen, 2017). This indicates that the role of size thresholds differs substantially between measures of tree recruitment that are based on recruitment of individual trees and overall recruited biomass. An explanation for this discrepancy is that recruited biomass aggregates over the processes growth, mortality, and recruitment of all trees, and therefore, only contains little information on the emergence of individual trees.

Differences in explanatory variables such as stem density and basal area only showed slight deviations of the effect estimates for the GER FR data set. But once both, explanatory and response variable, were based on the same diameter measurement threshold these deviations almost disappeared. There are two possible reasons for this finding: Either the high level of stochasticity in the process of tree recruitment or the probability of survival along with tree growth does not change on average between diameters of 4 and 12 cm. While stochasticity is introduced by the multitude of factors that drive tree recruitment at all stages relevant to regeneration in combination with errors caused by the sampling method (Shoemaker et al., 2020), survival of trees smaller than 12 cm may be subject to more direct interactions between individual trees and the environment. In this respect, future studies focusing on the effects of biotic and abiotic drivers on survival and growth of trees in smaller size classes could yield valuable insights into this important stage of forest population demography. Furthermore, if our finding that the same factors govern tree recruitment at different size classes is confirmed by other studies this could have consequences for the modeling and projection of tree recruitment because trade‐offs between measurement size thresholds and spatial coverage of empirical data may be less severe (cf. Clark et al., 1999).

### 4.5 Methodological considerations

We considered different ways to calculate recruitment rates (see Appendix S3: Section A4 and Figure A6) but finally decided to use the annual recruitment rate based on the abundance of tree recruitment per ha instead of per‐capita recruitment rates. The reason for this is that a prerequisite for calculating species‐specific per‐capita rates is the presence of adult trees for each species in the previous inventory (Kohyama et al., 2018). This was only the case for a fraction of species and plots across all data sets (Table A5). As a consequence, the decision between per‐capita and per‐area recruitment rates is also a question of data coverage regarding species and environmental conditions.

There are various approaches for modeling tree recruitment based on different data sets. One option is to use a joint data set to fit one general model. We decided against this because the harmonization of variables due to the different DBH measurement thresholds would result in a considerable loss of information (i.e., all information for trees smaller than a DBH of 12 cm would have had to be discarded; cf. Table 1). Another reason against this option is that the inventory designs differed substantially, particularly the widely different plot sizes resulted in varying observation errors (Král et al., 2010). While the DBH threshold can be increased artificially (see Figures A2 and A3), period length and plot size were dealt with by using model offsets. Sampling designs could be integrated by using inclusion probabilities that is, stratified sampling. However, this would either result in a considerable loss of information or require many model fits using bootstrap techniques (Nahorniak et al., 2015). To avoid loss of information or excessive computation times, we decided to keep the individual data sets separate.

For climatic variables, we considered using means of seasonal degree‐day sum and water balance over the inventory periods and include temporal trends in the study. But this was not useful because the time of recruitment cannot be related to a specific point in time with a specific climatic condition. In this context, it is important to consider that the time until a tree reaches a size that is considered recruitment and appears in an inventory can vary between few years and 100 years (Bigler, 2016). This issue becomes even more severe if size measurement thresholds are larger or vary between data sets. Additional problems are caused by the fact that recruitment aggregates over multiple subprocesses (dispersal, germination, growth, survival) where each process has different relationships to climate.

One major issue of this study is the quantification of soil and water‐related variables. We used modeled and interpolated soil data to quantify bucket size (Hengl et al., 2017), which resulted in many absolute bucket size values to be unrealistically high. Our motivation for using these data in spite of their high values was that the ranking of the values between plots and data sets is more important than their absolute values. However, the linear model resulted in highly uncertain effect estimates, confronting us with the difficulty of distinguishing between uncertainty resulting from a weak or missing linear effect of water relations on tree recruitment and uncertainty due to poor data quality. The quality of soil data is a major issue in environmental modeling that can outweigh climatic signals when analyzing relationships between vegetation and climate (Folberth et al., 2016; Román Dobarco et al., 2019). Another indication of poor soil data quality is a comparison that took place in a later stage of this study where a set of soil samples from an ongoing field campaign within the CH FR was compared with the SoilGrid250 based variables. This comparison showed there was almost no correlation for the calculated bucket size. The reason for such a weak correlation may be that (a) a single soil sample per plot is not representative of the entire plot area, or (b) the modeled soil variables from the SoilGrid250 data are inaccurate. The conclusion that water relations have no effect on tree recruitment appears invalid not only from a process‐based perspective (cf. Fernandez‐Illescas et al., 2001; Speich et al., 2018), but also when considering experimental studies on water relations and forest regeneration (Anderson et al., 2001; Aranda et al., 2012; Davis et al., 1999; De Groote et al., 2018; Facelli et al., 1999; Madsen, 1995) or studies on the relationship between species traits and site conditions (Niinemets & Valladares, 2006), which clearly show distinct effects of water relations on tree regeneration. In addition to these explanations of the large credible intervals for water household‐related variables, we acknowledge that the credible intervals estimated from our model are very large, but consistent. Thus, we consider our approach conservative (Matuschek et al., 2017).

Another issue is the nonlinear and complex nature of several processes that are important for tree recruitment. Although generalized linear mixed models (GLMMs) revealed distinct effects of stand structure and the degree‐day sum on tree recruitment, these models were not able to account for water relations and could not elucidate whether different levels of drought tolerance affect the relationship between tree recruitment and climate. In recent decades, GLMMs were used successfully for analyzing growth and mortality (Cailleret et al., 2017; Fortin et al., 2008). However, for the smallest size class and especially for modeling tree recruitment and regeneration over larger scales, our study showed that many processes are most likely dominated by complex relationships with feedback between stand structure and climate that cannot be formulated in GLMMs. Thus, for further large‐scale studies on tree recruitment‐environment interactions, we strongly recommend the use of alternatives to GLMMs, such as process‐based Bayesian approaches (Clark, 2003; Lines et al., 2020). The advantage of process‐based models is that they treat complex processes with feedbacks within the stand more explicitly. Thus, the sources of uncertainty might be easier to detect. Our approach is still valid as it shows that some relationships can be described with GLMMs but when it comes to climatic variables that are likely to change within the next decades a process‐based understanding is necessary to identify (a) sources of uncertainty (e.g., specific soil parameters, microclimate) or (b) the sensitivity of tree species to environmental conditions that were never observed. This is particularly important when attempting to deal with uncertainties regarding water relations and to overcome the lack of high‐quality soil information.

## 5 CONCLUSIONS

Shade tolerance and stand structure proved to be pivotal factors that determine tree recruitment at the stand level. In comparison to climatic and water‐related factors, there is a high degree of uncertainty regarding the role of drought tolerance for tree recruitment. In essence, this means that stand structure is suitable to describe general patterns of tree recruitment within forest ecosystems but larger‐scaled factors (i.e., climate and water availability) only show weak signals when analyzing tree recruitment with empirical models. All factors showed similar effects throughout all size classes represented in our data. Although temperature partly showed positive effects on tree recruitment, we could not disentangle the role of water relations in this respect. This has major implications for the projection of the development of forest ecosystems under future climate because the drivers of tree recruitment are only known on the local scale and remain poorly understood on larger spatial scales where variation in climate is high.

Although data sets from different regions that were collected based on different sampling protocols are usually quite heterogeneous, we conclude that the long‐term monitoring of managed and unmanaged forests provides a valuable source of information for a better understanding of highly stochastic ecological processes such as tree recruitment (Lindenmayer & Likens, 2018; Meyer, 2020). Particularly in the face of climate change, future research will rely on the availability of such data to improve our knowledge of forest stand dynamics at larger scales in time and space. One major advantage of using different data sets simultaneously is that confirmatory results facilitate drawing conclusions based on different data sets. Furthermore, we showed that heterogeneous data sets can be used to quantitatively confirm general and qualitatively known patterns of tree recruitment in different regions.

## CONFLICT OF INTEREST

The authors declare that there is no conflict of interest.

## AUTHOR CONTRIBUTIONS

**Yannek Käber:** Conceptualization (lead); Data curation (lead); Formal analysis (lead); Investigation (lead); Methodology (lead); Project administration (lead); Software (lead); Validation (lead); Visualization (lead); Writing‐original draft (lead); Writing‐review & editing (lead). **Peter Meyer:** Data curation (supporting); Resources (equal); Writing‐review & editing (supporting). **Jonas Stillhard:** Data curation (supporting); Resources (equal); Software (supporting); Writing‐review & editing (supporting). **Emiel De Lombaerde:** Resources (supporting); Writing‐review & editing (supporting). **Jürgen Zell:** Methodology (supporting); Writing‐review & editing (supporting). **Golo Stadelmann:** Data curation (supporting); Resources (equal); Writing‐review & editing (supporting). **Harald Bugmann:** Conceptualization (supporting); Formal analysis (supporting); Funding acquisition (lead); Investigation (supporting); Methodology (supporting); Project administration (equal); Resources (equal); Supervision (lead); Writing‐original draft (supporting); Writing‐review & editing (supporting). **Christof Bigler:** Conceptualization (supporting); Formal analysis (supporting); Investigation (supporting); Methodology (supporting); Supervision (lead); Validation (supporting); Visualization (supporting); Writing‐original draft (supporting); Writing‐review & editing (equal).

### OPEN RESEARCH BADGES

This article has earned an Open Materials Badge for making publicly available the components of the research methodology needed to reproduce the reported procedure and analysis. In case of acceptance, we plan to make the R scripts available via Zenodo or Dryad. All materials are available at https://doi.org/10.5281/zenodo.5119919.

## Supporting information

Appendix S1

Appendix S2

Appendix S3

## Data Availability

The R‐code used to create the content of this paper is archived in a zenodo.org repository (https://doi.org/10.5281/zenodo.5119919). Raw data are available from the Swiss Forest Reserve Network (Peter Brang; https://www.wsl.ch/en/wald/biodiversitaet‐naturschutz‐urwald/naturwaldreservate.html), the Forest Reserves of nort‐western Germany **(**Peter Meyer; https://www.nw‐fva.de/NwInfo/tablemap.jsp; https://www.nw‐fva.de/index.php?id=33), the Flemish National Forest Inventory (Leen Govaere; https://www.natuurenbos.be/beleid‐wetgeving/natuurbeheer/bosinventaris/contact) and the Swiss National Forest Inventory (Christoph Fischer; www.lfi.ch/kontakt/kontakt‐en.php).
