# Supplementary material for: Tree recruitment is determined by stand structure and shade tolerance with uncertain role of climate and water relations
Source: Ecol Evol. 2021 Aug 19;11(17):12182–203. doi: 10.1002/ece3.7984 (PMC8427579; doi:10.1002/ece3.7984)
Supplement: Supplementary file 1 — Appendix S1 [file ECE3-11-12182-s001.docx]

Appendix S1

# A1 Shade casting ability values

Table A1: Table of shade casting ability values with original values from Leuschner & Ellenberg (2017) and complemented values based on qualitative evaluation of species properties (indicated with *). The value for the genus was calculated based on the mean of the species within one genus.

| Genus | SCA |  | Species | SCA |
| --- | --- | --- | --- | --- |
| Abies | 5 |  | Abies alba | 5 |
| Acer | 4 |  | Acer campestre | 3 |
| Aesculus | 3 |  | Acer platanoides | 4 |
| Alnus | 3 |  | Alnus glutinosa | 3 |
| Betula | 1 |  | Betula pendula | 1 |
| Carpinus | 5 |  | Betula pubescens | 1 |
| Celtis | 4 |  | Carpinus betulus | 5 |
| Cornus | 3 |  | Castanea sativa | 3 |
| Corylus | 3 |  | Cornus mas* | 3 |
| Crataegus | 3 |  | Cornus sanguinea* | 3 |
| Euonymus | 3 |  | Crataegus laevigata* | 3 |
| Fagus | 5 |  | Crataegus monogyna* | 3 |
| Frangula | 2 |  | Fagus sylvatica | 5 |
| Fraxinus | 3 |  | Frangula alnus* | 2 |
| Ilex | 5 |  | Fraxinus excelsior | 3 |
| Juglans | 5 |  | Ilex aquifolium* | 5 |
| Larix | 1 |  | Pinus nigra | 1 |
| Liriodendron | 5 |  | Pinus sylvestris | 1 |
| Ostrya | 1 |  | Prunus avium | 3 |
| Picea | 4 |  | Prunus padus | 3 |
| Pinus | 1 |  | Prunus serotina* | 3 |
| Platanus | 4 |  | Prunus spinosa* | 3 |
| Populus | 2 |  | Quercus cerris | 3 |
| Prunus | 3 |  | Quercus petraea | 3 |
| Pseudotsuga | 4 |  | Quercus pubescens | 3 |
| Quercus | 3 |  | Quercus rubra* | 4 |
| Robinia | 1 |  | Salix aurita* | 2 |
| Rosa | 2 |  | Salix caprea* | 2 |
| Sambucus | 3 |  | Salix triandra* | 1 |
| Sequoiadendron | 5 |  | Salix x mollissima* | 2 |
| Sorbus | 3 |  | Sambucus nigra* | 3 |
| Taxus | 5 |  | Sambucus racemosa* | 3 |
| Thuja | 5 |  | Sorbus aria | 3 |
| Tilia | 4 |  | Sorbus aucuparia | 2 |
| Ulmus | 4 |  | Sorbus torminalis | 3 |
| Viburnum | 3 |  | Tilia cordata | 4 |
|  |  |  | Tilia platyphyllos | 4 |
|  |  |  | Ulmus glabra | 4 |
|  |  |  | Ulmus minor | 4 |
|  |  |  | Viburnum opulus | 3 |

# A2 Summary statistics


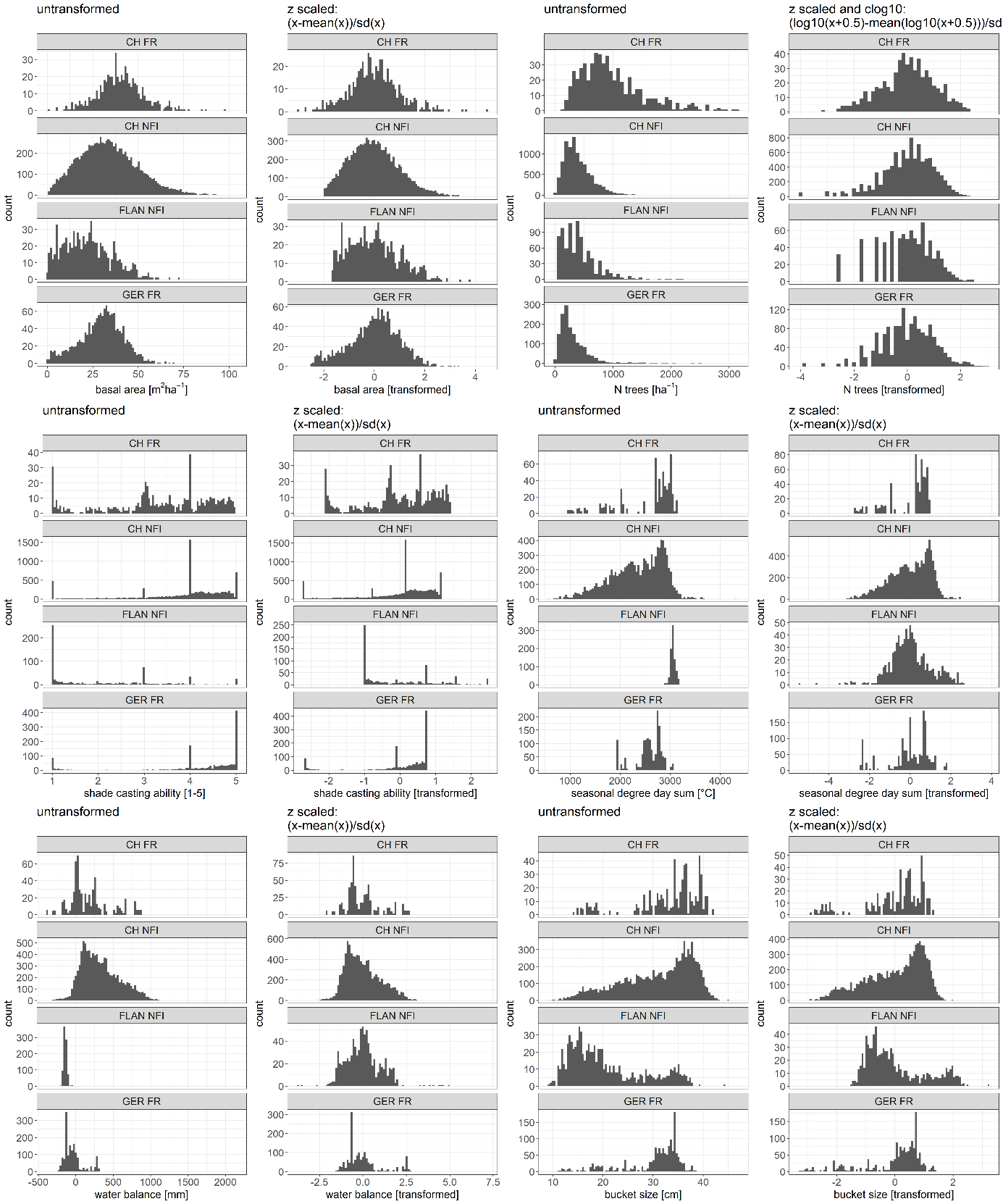
Figure A1: Histograms of original and standardized explanatory variables.

Table A2: Variance Inflation Factors (Naimi et al., 2014). *Values in brackets correspond to the Variance Inflation Factors for the GER FR data sets including water balance. The final model based on GER FR data did not include the water balance variable due to strong collinearity with degree day sum.

|  | Variance Inflation Factor | | | |
| --- | --- | --- | --- | --- |
| Variables | CH FR | GER FR | FLAN NFI | CH NFI |
| basal area | 1.45 | 1.56 (1.68)* | 2.34 | 1.53 |
| stem density | 1.26 | 1.83 (1.86)* | 2.20 | 1.47 |
| shade casting ability | 1.89 | 3.10 (3.05)* | 1.53 | 1.37 |
| degree day sum | 4.67 | 1.17 (14.16)* | 2.05 | 1.99 |
| water balance | 2.36 | - (14.62)* | 2.12 | 1.15 |
| bucket size | 2.67 | 2.94 (2.87)* | 1.43 | 2.25 |

Figure A2: Comparison of recruitment rates between artificially increased DBH thresholds and original data sets.


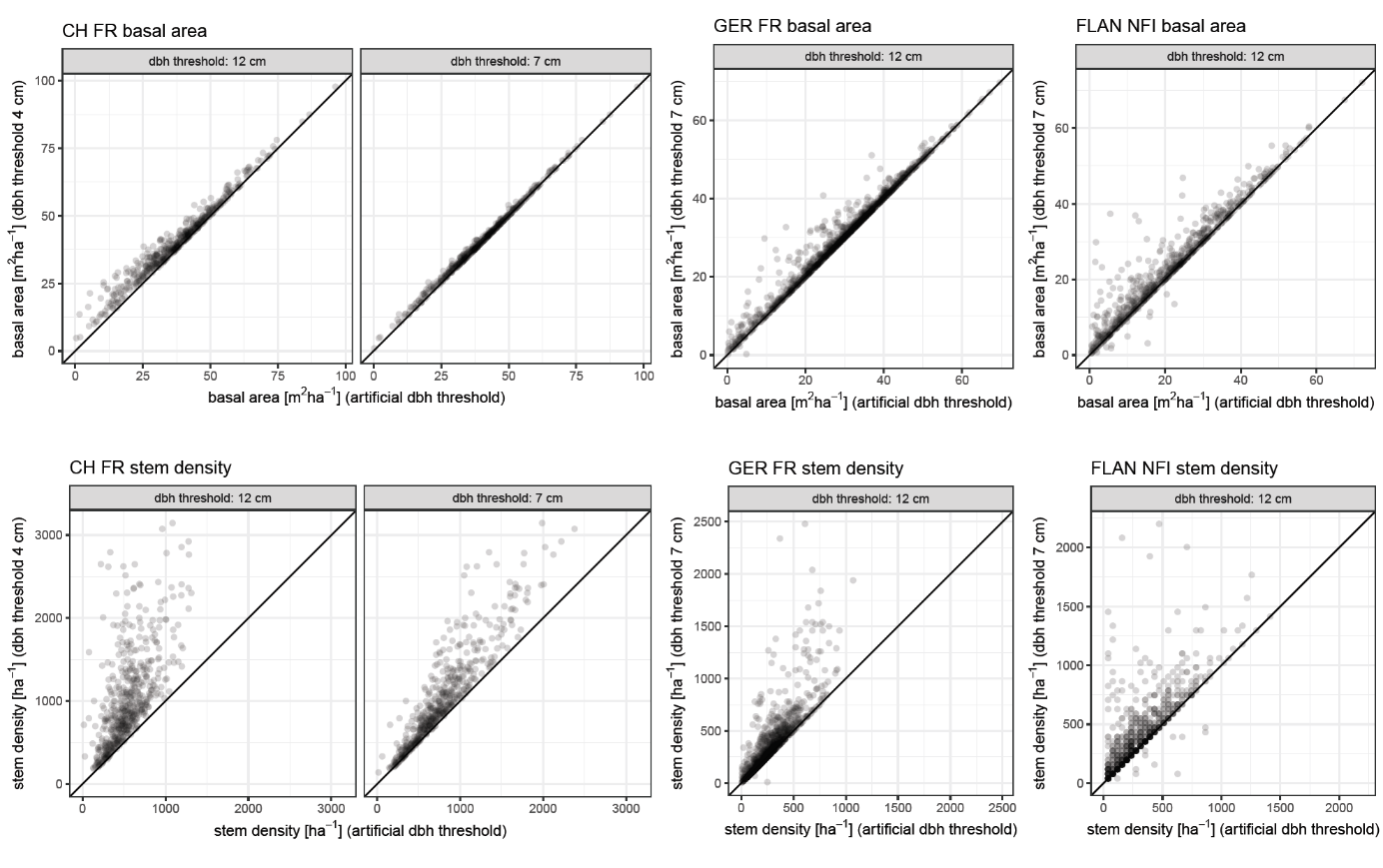


Figure A3: Comparison of explanatory variables stem basal area and stem density between artificially increased DBH thresholds and original data sets.


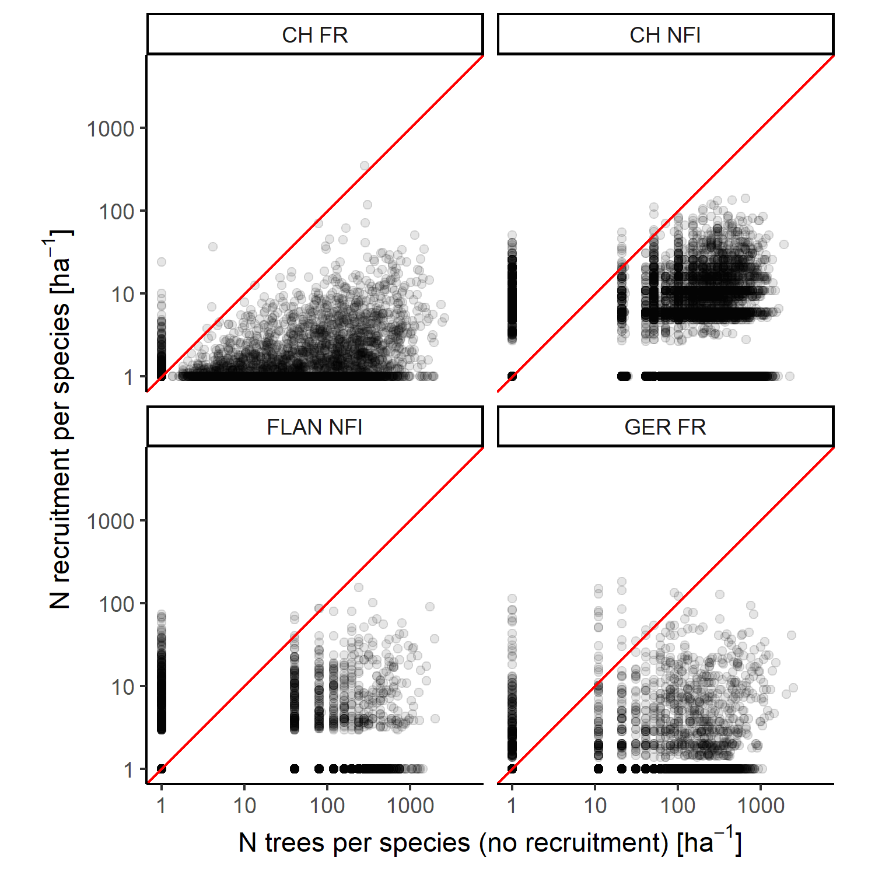


Figure A4: Abundance of trees per species that were observed for the first time at a plot (recruitment trees) vs. trees that were already present in the previous inventory. Note that both axes are transformed with log10(x+1).

Table A3: Percentage of total observed recruitment events where no conspecific adults where present in the previous inventory.

| species | CH FR | GER FR | FLAN NFI | CH NFI |
| --- | --- | --- | --- | --- |
| Abies alba | 8.1 | 0.0 |  | 21.1 |
| Acer campestre | 7.9 | 0.0 |  | 65.0 |
| Acer opalus | 5.6 |  |  | 78.6 |
| Acer platanoides | 25.9 | 50.0 |  | 33.3 |
| Acer pseudoplatanus | 14.8 | 47.1 | 33.3 | 54.2 |
| Alnus glutinosa | 5.6 |  | 27.6 | 47.6 |
| Alnus incana | 5.7 |  | 60.0 | 29.2 |
| Betula pendula | 18.8 |  |  | 33.1 |
| Carpinus betulus | 7.8 | 65.6 | 18.2 | 44.4 |
| Corylus avellana | 22.6 |  | 52.8 | 70.0 |
| Fagus sylvatica | 4.1 | 7.4 | 51.9 | 20.0 |
| Fraxinus excelsior | 9.9 | 43.1 | 37.0 | 48.2 |
| Ilex aquifolium | 50.0 | 53.9 |  | 76.9 |
| Juglans regia | 87.5 |  |  | 83.3 |
| Larix decidua | 7.1 |  |  |  |
| Picea abies | 1.9 | 15.4 | 43.8 | 9.5 |
| Pinus cembra | 0.0 |  |  | 12.7 |
| Pinus mugo | 0.0 |  |  | 8.6 |
| Pinus sylvestris | 3.6 | 8.6 | 10.2 | 9.1 |
| Populus | 42.9 |  | 25.0 | 47.4 |
| Prunus avium | 18.2 | 78.6 | 45.5 | 71.8 |
| Prunus padus | 3.3 |  |  | 83.3 |
| Prunus spinosa | 50.0 |  |  |  |
| Quercus | 4.7 | 36.5 | 38.7 | 30.8 |
| Robinia pseudoacacia | 30.0 |  | 0.0 | 57.1 |
| Salix | 16.4 | 70.8 | 45.0 | 60.4 |
| Sorbus aria | 6.7 |  |  | 70.6 |
| Sorbus aucuparia | 8.6 | 64.4 | 65.9 | 67.1 |
| Sorbus torminalis | 0.0 |  |  |  |
| Taxus baccata | 14.3 |  |  | 58.1 |
| Tilia cordata | 2.9 |  |  |  |
| Tilia platyphyllos | 42.9 | 40.0 |  | 50.0 |
| Ulmus glabra | 10.7 |  |  | 72.2 |
| Viburnum lantana | 55.6 |  |  |  |
| Viburnum opulus | 30.8 |  |  |  |
| Alnus |  | 0.0 |  |  |
| Betula |  | 25.5 |  |  |
| Larix |  | 66.7 | 16.7 | 14.5 |
| Pseudotsuga menziesii | |  | 33.3 | 50.0 |
| Ulmus |  | 20.0 | 20.0 |  |
| Betula tremula |  |  | 35.8 |  |
| Castanea sativa |  |  | 50.0 | 14.9 |
| Pinus nigra |  |  | 26.7 | 0.0 |
| Pinus strobus |  |  | 75.0 | 100.0 |
| Prunus serotina |  |  | 54.9 |  |
| Quercus rubra |  |  | 33.7 |  |
| Tilia |  |  | 66.7 |  |
| Betula pubescens | |  |  |  |
| Fraxinus ornus |  |  |  | 85.7 |
| Ostrya carpinifolia | |  |  |  |
| Tilia cordata |  |  |  | 45.3 |
| Ulmus minor |  |  |  | 100 |

# A3 Final composition of trait groups

Table A4: Species for each trait group and inventory. N denotes the total number of plot observations per data set. The percentage in brackets after each species refers to the proportion of total N per data set where at least one recruitment or adult tree of a species was observed.

|  | CH NFR (N = 574) | GER NFR (N = 1529) | FLAN NFI (N = 837) | CH NFI (N = 9939) |
| --- | --- | --- | --- | --- |
| D2S1 | *Betula pendula (18%), Salix (17%), Larix decidua (13%), Populus (10%),* | *Betula (12%), Larix (7%), Salix (4%), Populus (1%), Betula pendula (1%), Alnus (1%),* | *Betula tremula (30%), Populus (6%), Larix (4%), Salix (2%),* | *Larix (14%), Betula pendula (5%), Salix (1%), Populus (1%), Betula pubescens (<1%),* |
| D5S1 | *Pinus sylvestris (41%), Pinus mugo (15%), Robinia pseudoacacia (4%),* | *Pinus sylvestris (15%),* | *Pinus sylvestris (39%), Pinus nigra (11%), Robinia pseudoacacia (1%),* | *Pinus sylvestris (10%), Pinus mugo (2%), Robinia pseudoacacia (<1%), Pinus nigra (<1%), Fraxinus ornus (<1%),* |
| D2S3 | *Fraxinus excelsior (58%), Sorbus aucuparia (17%), Alnus incana (13%), Alnus glutinosa (9%), Prunus padus (6%), Viburnum opulus (3%),* | *Fraxinus excelsior (13%), Sorbus aucuparia (6%), Pinus strobus (1%), Pseudotsuga menziesii (1%), Alnus glutinosa (1%),* | *Alnus glutinosa (8%), Sorbus aucuparia (6%), Fraxinus excelsior (5%), Pseudotsuga menziesii (1%), Alnus incana (1%), Pinus strobus (<1%),* | *Fraxinus excelsior (15%), Alnus incana (3%), Sorbus aucuparia (2%), Alnus glutinosa (1%), Pseudotsuga menziesii (1%), Pinus strobus (<1%), Prunus padus (<1%),* |
| D4S3 | *Quercus (51%), Sorbus aria (39%), Sorbus torminalis (5%), Juglans regia (5%), Pinus cembra (4%), Prunus spinosa (2%), Viburnum lantana (2%),* | *Quercus (29%),* | *Quercus (35%), Quercus rubra (16%), Prunus serotina (8%), Castanea sativa (6%),* | *Quercus (9%), Castanea sativa (3%), Sorbus aria (3%), Pinus cembra (2%), Juglans regia (<1%), Sorbus torminalis (<1%), Ulmus minor (<1%),* |
| D3S4 | *Acer pseudoplatanus (57%), Tilia cordata (39%), Corylus avellana (29%), Prunus avium (29%), Acer campestre (26%), Acer platanoides (26%), Ulmus glabra (25%), Carpinus betulus (23%), Taxus baccata (14%), Ilex aquifolium (10%), Acer opalus (8%), Tilia platyphyllos (2%),* | *Acer pseudoplatanus (16%), Carpinus betulus (8%), Acer platanoides (5%), Ulmus (4%), Ilex aquifolium (2%), Prunus avium (2%), Tilia platyphyllos (1%), Acer campestre (1%), Tilia cordata (<1%),* | *Acer pseudoplatanus (8%), Corylus avellana (4%), Carpinus betulus (2%), Prunus avium (2%), Ulmus (1%), Tilia (1%),* | *Acer pseudoplatanus (17%), Prunus avium (3%), Ulmus glabra (2%), Tilia cordata (2%), Carpinus betulus (2%), Acer platanoides (1%), Tilia platyphyllos (1%), Acer campestre (1%), Taxus baccata (1%), Acer opalus (1%), Ostrya carpinifolia (1%), Corylus avellana (<1%), Ilex aquifolium (<1%),* |
| D2S5 | *Fagus sylvatica (65%), Picea abies (63%), Abies alba (36%),* | *Fagus sylvatica (79%), Picea abies (35%), Abies alba (1%),* | *Fagus sylvatica (7%), Picea abies (3%),* | *Picea abies (66%), Fagus sylvatica (46%), Abies alba (33%),* |


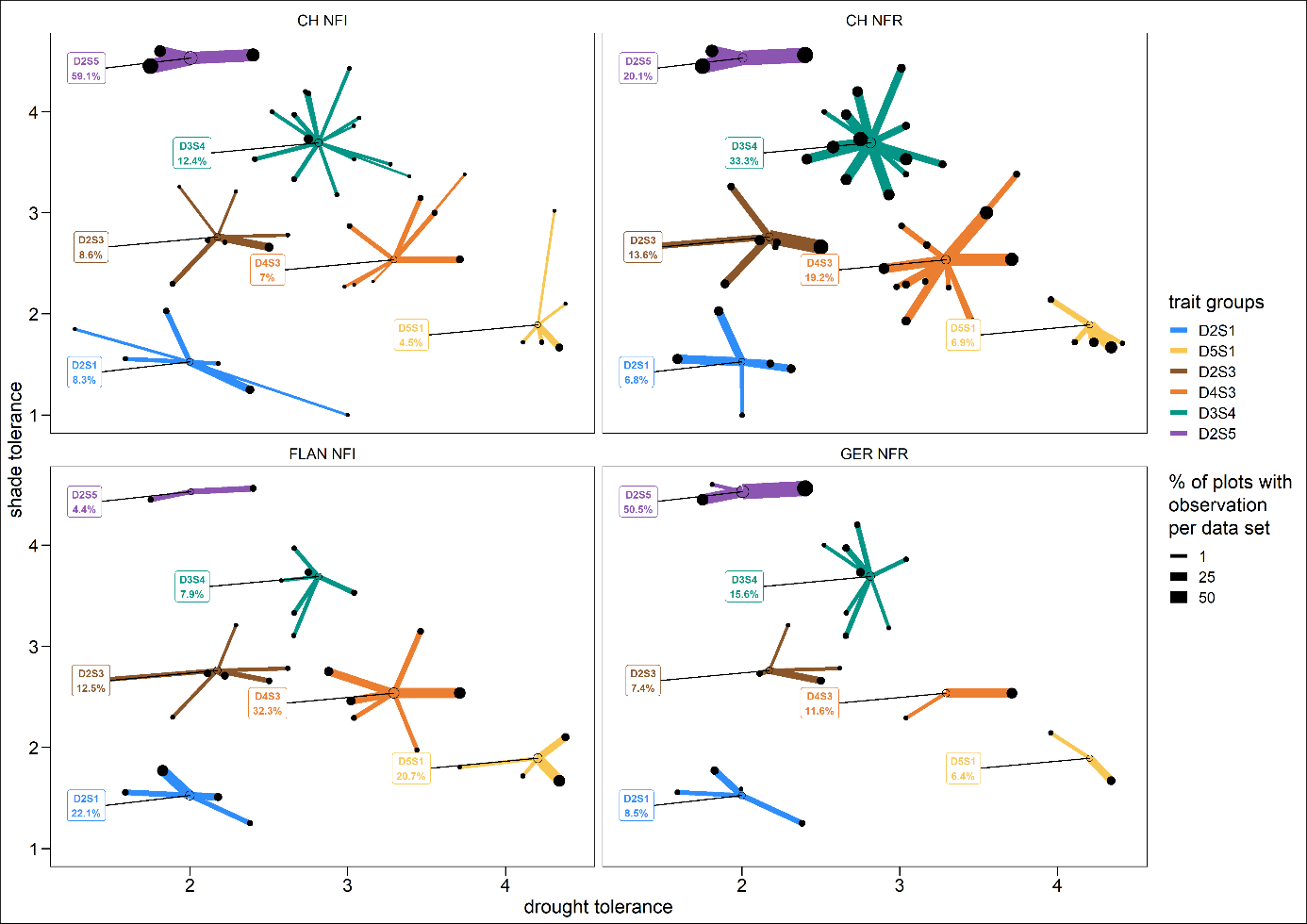


Figure A5: Results of clustering species in trait groups for each individual data set. Black points refer to individual tree species. The thickness of a line refers to the fraction of the plots in which a species was observed. The coloured boxes inside the panels refer to the percentage contribution of a trait group to the data set.

The distribution of the six species groups is most balanced in the Swiss NFRs, while the absolute number of observations is highest in the Swiss NFI. The Flan NFI has a large fraction of trait group D4S3 (*Quercus*) and trait group D5S1 (*Pinus sylvestris*) compared to the other data sets, which are more dominated by trait group D2S5. Table A2 lists all species and its corresponding trait group. Figure A2 illustrates the species composition of the trait groups for each individual data set. Generally, the distribution of species across the trait groups resulted in sufficient observations to estimate model coefficients for all trait groups, and no information on the species level was lost. The fraction of plot observations with at least one occurring tree for each tree species, including tree recruitment and adult trees, in each data set ranges from less than 1% to almost 80% of all plot observations.

Trait group D2S1, which characterizes light-demanding and drought intolerant species, is mostly composed of *Betula* spp. and *Larix* spp.. The share of all observations per data set for the most abundant species is between 12 % (Ger NFR) and 30 % (Flan NFI) and indicates good coverage across all data sets.

Trait group D5S1, which consists of very light-demanding and drought-adapted species, is mostly composed of *Pinus sylvestris*, *Pinus mugo* and *Pinus nigra*. In addition to *Pinus* spp., a small fraction of *Robinia pseudoacacia* and *Fraxinus ornus* is also represented. The share of all observations per data set for the most abundant species (*Pinus sylvestris*) ranges from 10 % (Swiss NFI) and 15 % (Ger NFR) to 39 % (Flan NFI) and 41 % (Swiss NFR).

Trait group D2S3, which represents moderately shade-tolerant and drought-intolerant species, consists mostly of *Fraxinus excelsior* with some *Sorbus aucuparia*, *Prunus padus* and *Alnus* spp.*.* Note that this group also contains *Pinus strobus* in the Swiss data sets. The share of all observations per data set for the most abundant species (*Fraxinus excelsior)* ranges from 5 % (Flan NFI) to 58 % (Swiss NFR), whereas *Alnus glutinosa* is the most abundant species in the Flan NFI with a share of 8 % from all observations.

Trait group D4S3, which represents moderately shade-tolerant and drought-tolerant species, consists mostly of *Quercus* spp. with *Sorbus* spp., *Prunus* spp. and *Castanea sativa*. Note that this group also contains *Pinus cembra* in the Swiss data sets. The share of all observations per data set for the most abundant species (*Quercus* spp.*)* ranges from 9 % (Swiss NFI) to 51 % (Swiss NFR).

Trait group D3S4, which characterizes shade-tolerant species with moderate adaption to drought, is dominated by *Acer pseudoplatanus*. Additionally, *Tilia cordata*, *Corylus avelana*, *Prunus avium*, *Acer* and *Ulmus* species are in this group. Given the number of species that are abundant in this group. The group is generally well represented.

Trait group D2S5, which characterizes very shade-tolerant and drought-intolerant species, is composed of *Fagus sylvatica*, *Picea abies* and *Abies alba*. Except for the Flan NFI, this group has the largest share of observations per data set, whereas it is the least represented in the Flan NFI.

# A4 Abundance of tree recruitment vs. recruitment rates

Modelling the total number of recruitment trees observed on a reassessed forest inventory plot comes with the disadvantage of not accounting for recruited trees that die within one inventory period. Calculating recruitment rates relative to the population is a possible solution to deal with such a bias (Kohyama et al., 2018). We decided against the calculation of such recruitment rates for two reasons. First, a prerequisite for calculating recruitment rates based on previous populations is that the species or the (sub)population of interest is traceable. This means that at least one surviving individual per species must exist at the time of re-assessment of the plots. As a result, many observations of certain species would have been excluded with a considerable loss of information within the available data sets (Table A5). Either in terms of species or coverage of environmental gradients. Second, our aim was to analyse differences between successional groups, not the calculation of the most accurate recruitment rates *per se*. We therefore wish to point out that the modelled number of recruited trees within a certain period should not be interpreted as annual recruitment rate, but as the probability of observing new individuals of a certain successional group in a population. This was a compromise between not losing information and obtaining a model that allows for ecological inference. Moreover, we defined trait groups to save species-specific information on tolerance to shade and drought within all data sets. For dynamic modelling, however, we recommend using the annual recruitment rates described by Kohyama et al. (2018) when dealing with inventory based records of tree recruitment. The differences between such rates and abundance of tree recruitment used in this study is illustrated in Figure A1 with a comparison between final-density-based recruitment rates (Table 1, eq. 7 in Kohyama et al., 2018) and the annual recruitment rates per area used for this study.


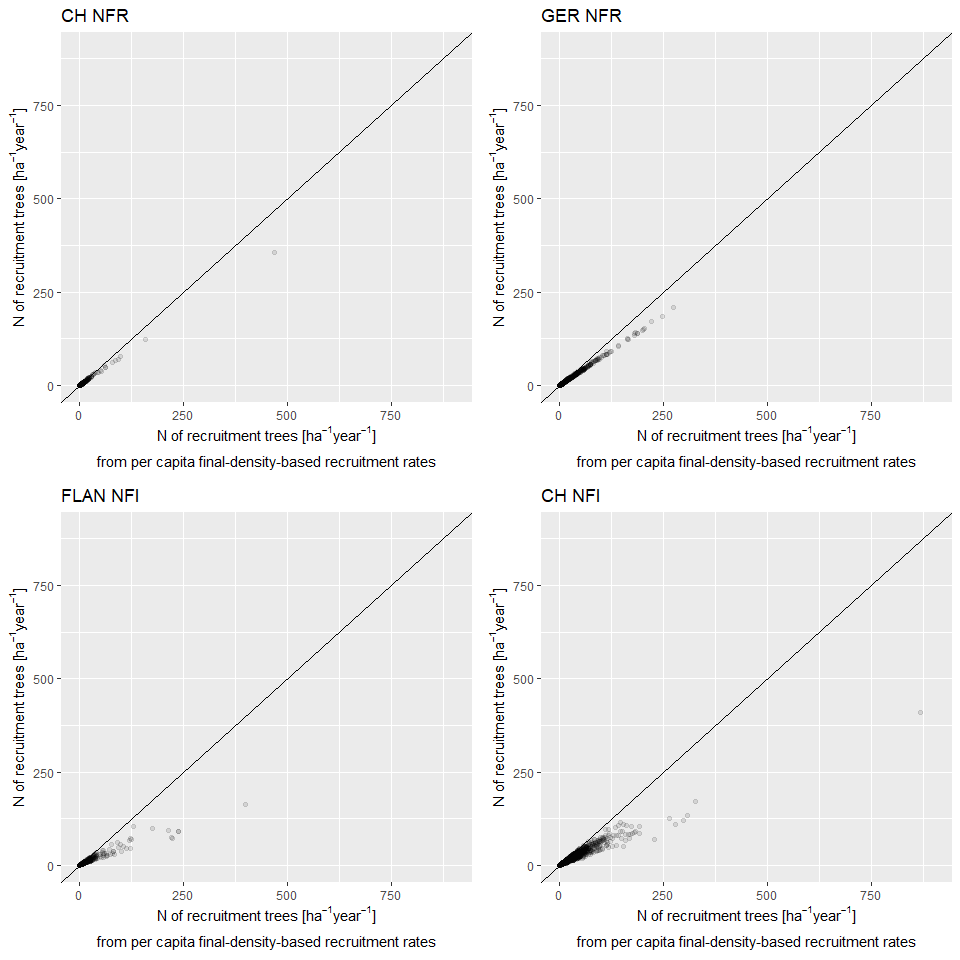


Figure A6: Comparison between N of recruitment trees $[{ha}^{-1} {year}^{-1}]$ and N of recruitment trees $[{ha}^{-1} {year}^{-1}]$ calculated as final stem density multiplied by the final-density per capita recruitment rate as described in eq. 7 in Kohyama et al. (2018). This figure illustrates the difference between recruitment calculated from rates based on a tracked subpopulation on the x-axis (i.e. species that occurred at least once in consecutive inventories) and recruitment of a species which could also be absent in previous inventories on the y-axis.

Table A5: Percentage of total observed recruitment events where no conspecific adults were present in the previous inventory.

| species | CH FR | GER FR | FLAN NFI | CH NFI |
| --- | --- | --- | --- | --- |
| Abies alba | 8.1 | 0.0 |  | 21.1 |
| Acer campestre | 7.9 | 0.0 |  | 65.0 |
| Acer opalus | 5.6 |  |  | 78.6 |
| Acer platanoides | 25.9 | 50.0 |  | 33.3 |
| Acer pseudoplatanus | 14.8 | 47.1 | 33.3 | 54.2 |
| Alnus glutinosa | 5.6 |  | 27.6 | 47.6 |
| Alnus incana | 5.7 |  | 60.0 | 29.2 |
| Betula pendula | 18.8 |  |  | 33.1 |
| Carpinus betulus | 7.8 | 65.6 | 18.2 | 44.4 |
| Corylus avellana | 22.6 |  | 52.8 | 70.0 |
| Fagus sylvatica | 4.1 | 7.4 | 51.9 | 20.0 |
| Fraxinus excelsior | 9.9 | 43.1 | 37.0 | 48.2 |
| Ilex aquifolium | 50.0 | 53.9 |  | 76.9 |
| Juglans regia | 87.5 |  |  | 83.3 |
| Larix decidua | 7.1 |  |  |  |
| Picea abies | 1.9 | 15.4 | 43.8 | 9.5 |
| Pinus cembra | 0.0 |  |  | 12.7 |
| Pinus mugo | 0.0 |  |  | 8.6 |
| Pinus sylvestris | 3.6 | 8.6 | 10.2 | 9.1 |
| Populus | 42.9 |  | 25.0 | 47.4 |
| Prunus avium | 18.2 | 78.6 | 45.5 | 71.8 |
| Prunus padus | 3.3 |  |  | 83.3 |
| Prunus spinosa | 50.0 |  |  |  |
| Quercus | 4.7 | 36.5 | 38.7 | 30.8 |
| Robinia pseudoacacia | 30.0 |  | 0.0 | 57.1 |
| Salix | 16.4 | 70.8 | 45.0 | 60.4 |
| Sorbus aria | 6.7 |  |  | 70.6 |
| Sorbus aucuparia | 8.6 | 64.4 | 65.9 | 67.1 |
| Sorbus torminalis | 0.0 |  |  |  |
| Taxus baccata | 14.3 |  |  | 58.1 |
| Tilia cordata | 2.9 |  |  |  |
| Tilia platyphyllos | 42.9 | 40.0 |  | 50.0 |
| Ulmus glabra | 10.7 |  |  | 72.2 |
| Viburnum lantana | 55.6 |  |  |  |
| Viburnum opulus | 30.8 |  |  |  |
| Alnus |  | 0.0 |  |  |
| Betula |  | 25.5 |  |  |
| Larix |  | 66.7 | 16.7 | 14.5 |
| Pseudotsuga menziesii | |  | 33.3 | 50.0 |
| Ulmus |  | 20.0 | 20.0 |  |
| Betula tremula |  |  | 35.8 |  |
| Castanea sativa |  |  | 50.0 | 14.9 |
| Pinus nigra |  |  | 26.7 | 0.0 |
| Pinus strobus |  |  | 75.0 | 100.0 |
| Prunus serotina |  |  | 54.9 |  |
| Quercus rubra |  |  | 33.7 |  |
| Tilia |  |  | 66.7 |  |
| Betula pubescens | |  |  |  |
| Fraxinus ornus |  |  |  | 85.7 |
| Ostrya carpinifolia | |  |  |  |
| Tilia cordata |  |  |  | 45.3 |
| Ulmus minor |  |  |  | 100 |
