# Supplementary material for: Tree recruitment is determined by stand structure and shade tolerance with uncertain role of climate and water relations
Source: Ecol Evol. 2021 Aug 19;11(17):12182–203. doi: 10.1002/ece3.7984 (PMC8427579; doi:10.1002/ece3.7984)
Supplement: Supplementary file 2 — Appendix S2 [file ECE3-11-12182-s003.docx]

Appendix S2

# B1 Model R-Code

The models were implemented with the brms package in the following way for all data sets. Only the water balance was not included for the GER FR data set.

For more details see the supplementary materials (placeholder for link) but note that the original code has slightly different variable names because the variable names were changed here to make its meaning clearer.

# **B2 Model diagnostic**

#
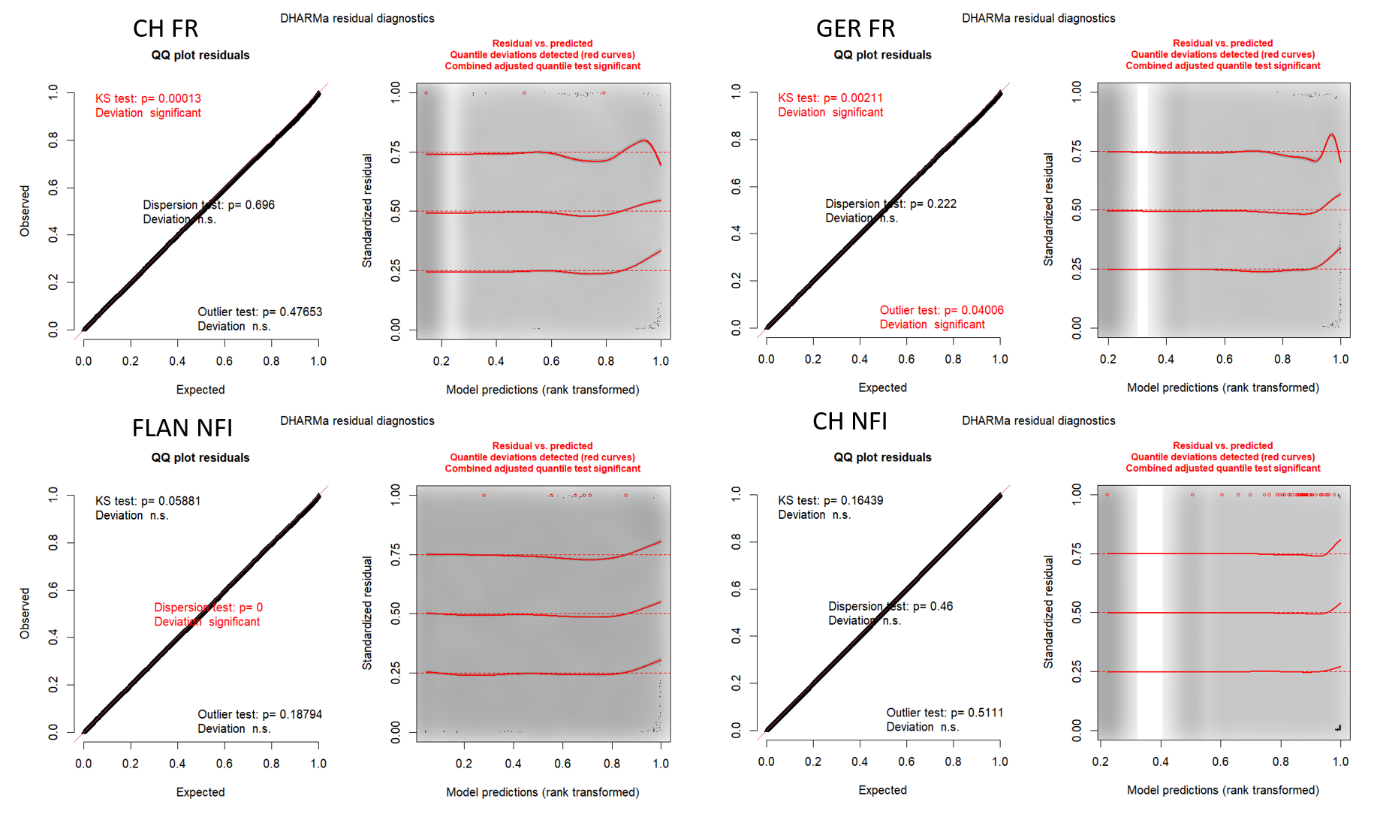


Figure B1: Scaled residuals based on simulated residuals (Hartig, 2020). Patterns at higher values may arise from site random effects but also suggest that the models are overestimating high abundance of tree recruitment.


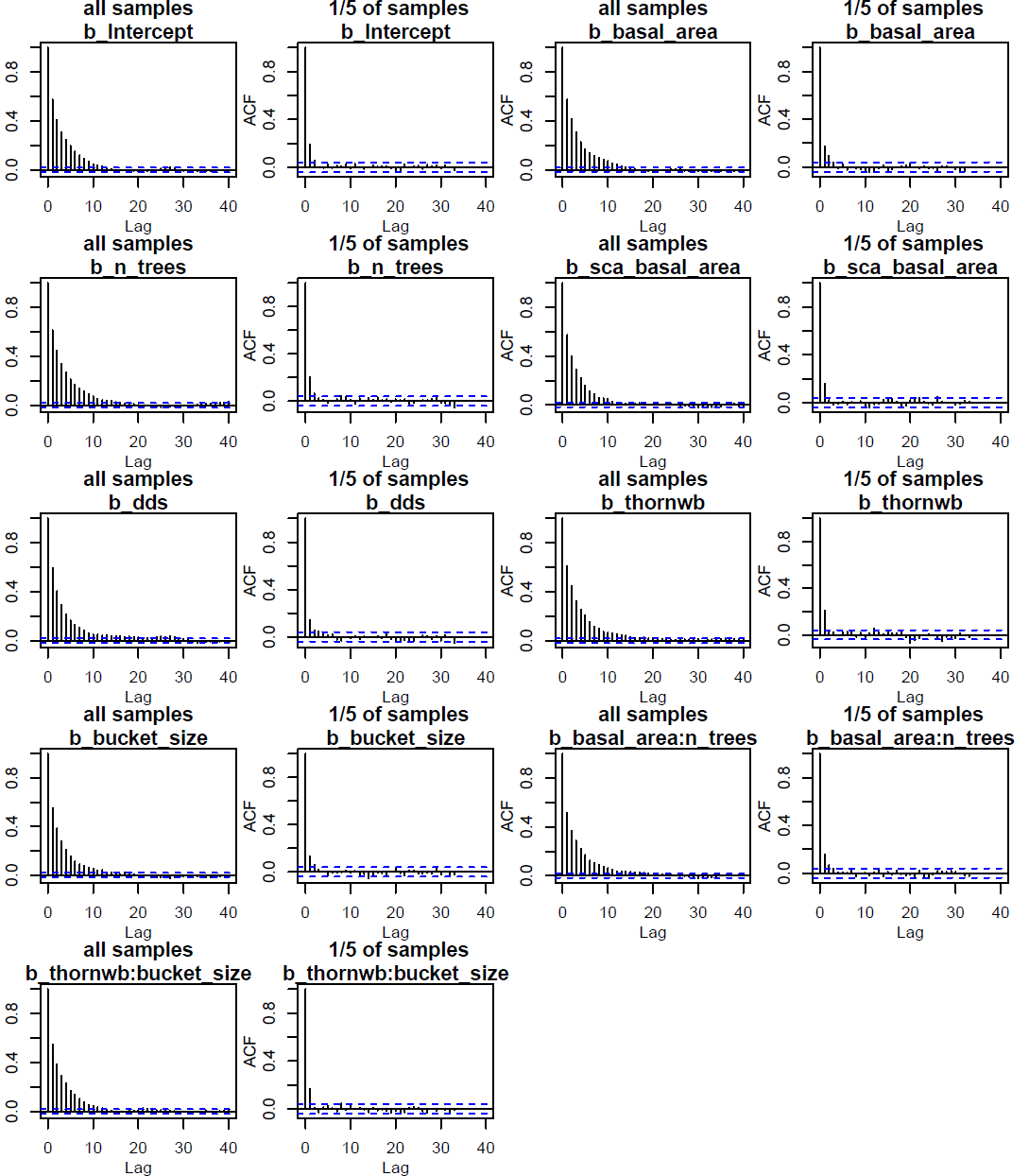


Figure B2: Autocorrelation for the main effects of the CH FR model for all MCMC samples and for 1/5th of all samples. This example of thinning the MCMC samples for the main effects and the CH FR model applied to all parameters and models.

# B3 Model results summary

Table B1: Estimated model coefficients for each data set including 95 % credible intervals. Trait group D2S5 is used as reference level.

|  | **CH FR** | | **GER FR** | | **FLAN NFI** | | **CH NFI** | |
| --- | --- | --- | --- | --- | --- | --- | --- | --- |
| Predictors | Log-Mean | CI (95%) | Log-Mean | CI (95%) | Log-Mean | CI (95%) | Log-Mean | CI (95%) |
| Intercept | -2.37 | -5.67 – 1.12 | -6.12 | -10.11 – -2.72 | -3.49 | -6.70 – -0.30 | -0.62 | -2.94 – 1.73 |
| basal area | -0.36 | -1.28 – 0.60 | -0.66 | -1.27 – 0.18 | -0.31 | -1.23 – 0.61 | -0.82 | -1.61 – -0.04 |
| stem density | 0.18 | -1.23 – 1.60 | 0.69 | 0.02 – 1.30 | 1.13 | 0.28 – 1.98 | 0.45 | -0.28 – 1.19 |
| shade casting ability | 1.6 | 0.21 – 2.92 | 0.14 | -1.49 – 1.72 | 1.41 | 0.47 – 2.33 | 0.57 | 0.09 – 1.06 |
| degree day sum | 1.11 | -2.66 – 4.80 | 0.53 | -1.76 – 3.13 | 0.41 | -0.39 – 1.25 | 0.01 | -1.08 – 1.08 |
| water balance | 0.56 | -1.86 – 2.83 |  |  | 0.31 | -0.52 – 1.19 | 0.04 | -0.68 – 0.78 |
| bucket size | 0.34 | -1.51 – 2.17 | -0.91 | -3.20 – 1.33 | -0.11 | -1.51 – 1.34 | 0.3 | -0.46 – 1.08 |
| D2S1 | -5.62 | -10.31 – -1.12 | -2.36 | -6.79 – 2.80 | -0.05 | -4.19 – 3.81 | -4.89 | -8.01 – -1.89 |
| D5S1 | -8.88 | -14.11 – -4.10 | -10.56 | -17.98 – -2.47 | -1.68 | -5.85 – 2.38 | -7.4 | -10.56 – -4.35 |
| D2S3 | -4.24 | -8.46 – -0.24 | -1.58 | -6.28 – 3.72 | 0 | -3.78 – 3.55 | -5.1 | -8.03 – -2.27 |
| D4S3 | -6.12 | -10.38 – -2.15 | -1.72 | -8.57 – 5.88 | 2.7 | -1.15 – 6.54 | -5.77 | -8.74 – -2.91 |
| D3S4 | -4.33 | -8.13 – -0.63 | -2.29 | -6.31 – 2.46 | -0.83 | -4.52 – 2.83 | -5.29 | -7.90 – -2.71 |
| basal_area.stem density | 0.03 | -0.49 – 0.53 | 0.58 | 0.05 – 1.12 | 0.11 | -0.43 – 0.67 | 0.12 | -0.09 – 0.33 |
| water balance.bucket size | -0.6 | -1.92 – 0.66 |  |  | -0.29 | -0.89 – 0.27 | -0.02 | -0.41 – 0.38 |
| basal_area.D2S1 | -1.46 | -2.78 – -0.18 | -1.07 | -2.09 – -0.25 | -1.13 | -2.27 – -0.04 | -0.39 | -1.38 – 0.61 |
| basal area.D5S1 | -1.56 | -2.95 – -0.17 | -3.93 | -5.52 – -2.54 | -1.62 | -2.81 – -0.40 | -1.05 | -2.14 – 0.04 |
| basal area.D2S3 | -0.36 | -1.51 – 0.80 | -0.27 | -1.29 – 0.59 | -0.79 | -1.88 – 0.26 | -0.74 | -1.71 – 0.24 |
| basal area.D4S3 | -0.52 | -1.64 – 0.61 | 0.1 | -1.41 – 1.36 | -0.08 | -1.12 – 0.98 | -0.14 | -1.12 – 0.87 |
| basal area.D3S4 | -0.22 | -1.27 – 0.81 | 0.46 | -0.41 – 1.19 | -1.05 | -2.11 – 0.07 | -0.32 | -1.22 – 0.53 |
| stem density.D2S1 | -0.52 | -2.51 – 1.40 | -0.21 | -1.01 – 0.67 | -0.47 | -1.51 – 0.58 | 0.03 | -0.94 – 1.00 |
| stem density.D5S1 | 0.13 | -2.00 – 2.24 | 1.49 | 0.27 – 2.71 | 0.9 | -0.17 – 2.02 | 0.65 | -0.38 – 1.72 |
| stem density.D2S3 | -1.08 | -2.85 – 0.65 | -0.22 | -1.06 – 0.63 | -0.71 | -1.74 – 0.25 | 0.29 | -0.64 – 1.23 |
| stem density.D4S3 | 0.61 | -1.12 – 2.36 | -0.3 | -1.48 – 0.87 | -0.57 | -1.58 – 0.39 | 0.18 | -0.77 – 1.13 |
| stem density.D3S4 | 0.84 | -0.71 – 2.39 | -0.08 | -0.77 – 0.67 | -0.41 | -1.42 – 0.59 | 0.19 | -0.62 – 1.04 |
| shade casting ability.D2S1 | -2.6 | -4.43 – -0.80 | -1.59 | -3.71 – 0.55 | -2.4 | -3.60 – -1.21 | -1.26 | -1.90 – -0.63 |
| shade casting ability.D5S1 | -4.64 | -6.59 – -2.55 | -5.02 | -8.48 – -1.71 | -2.85 | -4.17 – -1.53 | -1.71 | -2.39 – -1.07 |
| shade casting ability.D2S3 | -2.73 | -4.40 – -1.05 | -1.24 | -3.53 – 1.07 | -1.59 | -2.69 – -0.51 | -0.73 | -1.33 – -0.13 |
| shade casting ability.D4S3 | -2.83 | -4.46 – -1.15 | -2.13 | -5.25 – 0.94 | -1.54 | -2.67 – -0.42 | -1.28 | -1.91 – -0.65 |
| shade casting ability.D3S4 | -0.6 | -2.11 – 0.89 | -1.41 | -3.25 – 0.55 | -0.74 | -1.85 – 0.34 | -0.77 | -1.31 – -0.22 |
| degree day sum.D2S1 | 0.26 | -4.63 – 5.32 | 0.09 | -3.10 – 3.04 | -0.24 | -1.25 – 0.78 | -0.02 | -1.41 – 1.38 |
| degree day sum.D5S1 | -0.01 | -5.52 – 5.61 | 3.55 | -1.53 – 8.29 | -0.49 | -1.60 – 0.62 | 0.51 | -0.88 – 1.92 |
| degree day sum.D2S3 | 4.48 | 0.06 – 9.31 | 0.01 | -3.16 – 3.21 | 0.02 | -1.01 – 0.94 | 0.51 | -0.84 – 1.84 |
| degree day sum.D4S3 | 1.34 | -3.19 – 5.93 | 0.32 | -4.50 – 4.78 | -0.63 | -1.64 – 0.29 | 0.58 | -0.76 – 1.97 |
| degree day sum.D3S4 | 3.79 | -0.37 – 8.03 | 0.36 | -2.48 – 3.07 | -0.15 | -1.11 – 0.77 | 1.08 | -0.10 – 2.30 |
| water balance.D2S1 | -0.56 | -3.66 – 2.66 |  |  | -0.81 | -2.02 – 0.20 | 0.1 | -0.84 – 1.04 |
| water balance.D5S1 | -1.34 | -4.80 – 2.08 |  |  | -0.86 | -2.03 – 0.25 | -0.09 | -1.07 – 0.84 |
| water balance.D2S3 | 1.73 | -1.05 – 4.61 |  |  | 0.1 | -0.93 – 1.14 | -0.05 | -0.97 – 0.84 |
| water balance.D4S3 | 0.36 | -2.42 – 3.16 |  |  | -0.94 | -1.99 – 0.07 | 0.21 | -0.77 – 1.12 |
| water balance.D3S4 | 0.19 | -2.41 – 2.79 |  |  | -0.52 | -1.59 – 0.48 | 0.04 | -0.79 – 0.84 |
| bucket size.D2S1 | -0.93 | -3.42 – 1.51 | 1.76 | -1.11 – 4.90 | 0 | -1.79 – 1.69 | -0.6 | -1.61 – 0.43 |
| bucket size.D5S1 | -0.63 | -3.40 – 2.20 | 2.12 | -2.40 – 6.69 | -1.72 | -3.68 – 0.21 | -0.12 | -1.20 – 0.94 |
| bucket size.D2S3 | 0.24 | -1.99 – 2.53 | 2.23 | -0.86 – 5.43 | 0.05 | -1.68 – 1.65 | 0.22 | -0.74 – 1.21 |
| bucket size.D4S3 | 0.32 | -1.85 – 2.57 | 0.42 | -3.99 – 4.90 | -0.19 | -1.95 – 1.53 | -0.36 | -1.38 – 0.64 |
| bucket size.D3S4 | 0.32 | -1.66 – 2.39 | 3.46 | 0.83 – 6.17 | 1.08 | -0.61 – 2.71 | 0.25 | -0.61 – 1.13 |
| basal area.stem density.D2S1 | 0.17 | -0.53 – 0.92 | -0.51 | -1.24 – 0.14 | -0.04 | -0.70 – 0.67 | 0.04 | -0.24 – 0.34 |
| basal area.stem density.D5S1 | 0.09 | -0.72 – 0.91 | 0.97 | -0.05 – 2.04 | 0.52 | -0.23 – 1.22 | 0.08 | -0.28 – 0.43 |
| basal area.stem density.D2S3 | -0.09 | -0.74 – 0.55 | -0.47 | -1.21 – 0.22 | -0.11 | -0.76 – 0.52 | 0 | -0.28 – 0.27 |
| basal area.stem density.D4S3 | 0.37 | -0.27 – 1.04 | -0.81 | -1.87 – 0.22 | 0.02 | -0.60 – 0.64 | 0.06 | -0.24 – 0.34 |
| basal area.stem density.D3S4 | 0.39 | -0.14 – 0.97 | -0.67 | -1.31 – -0.08 | -0.14 | -0.83 – 0.49 | -0.05 | -0.30 – 0.21 |
| water balance.bucket size.D2S1 | -0.05 | -1.73 – 1.76 |  |  | 0.28 | -0.39 – 1.01 | 0.04 | -0.47 – 0.59 |
| water balance.bucket size.D5S1 | 0.95 | -1.02 – 3.04 |  |  | -0.8 | -1.64 – 0.05 | 0.29 | -0.26 – 0.86 |
| water balance.bucket size.D2S3 | 1.51 | -0.06 – 3.17 |  |  | 0.01 | -0.73 – 0.65 | 0.05 | -0.45 – 0.57 |
| water balance.bucket size.D4S3 | 1.68 | 0.15 – 3.30 |  |  | 0.06 | -0.63 – 0.73 | 0.04 | -0.48 – 0.58 |
| water balance.bucket size.D3S4 | 1.18 | -0.17 – 2.66 |  |  | 0.42 | -0.23 – 1.07 | 0.23 | -0.22 – 0.70 |
| N | 258 _area_plot_id_ | | 1320 _area_plot_id_ | | 837 _plot_id_ | | 4519 _plot_id_ | |
|  | 35 _species_ | | 20 _species_ | | 25 _species_ | | 40 _species_ | |
| Observations | 20090 | | 30580 | | 20925 | | 397560 | |

# B4 Additional simulation results


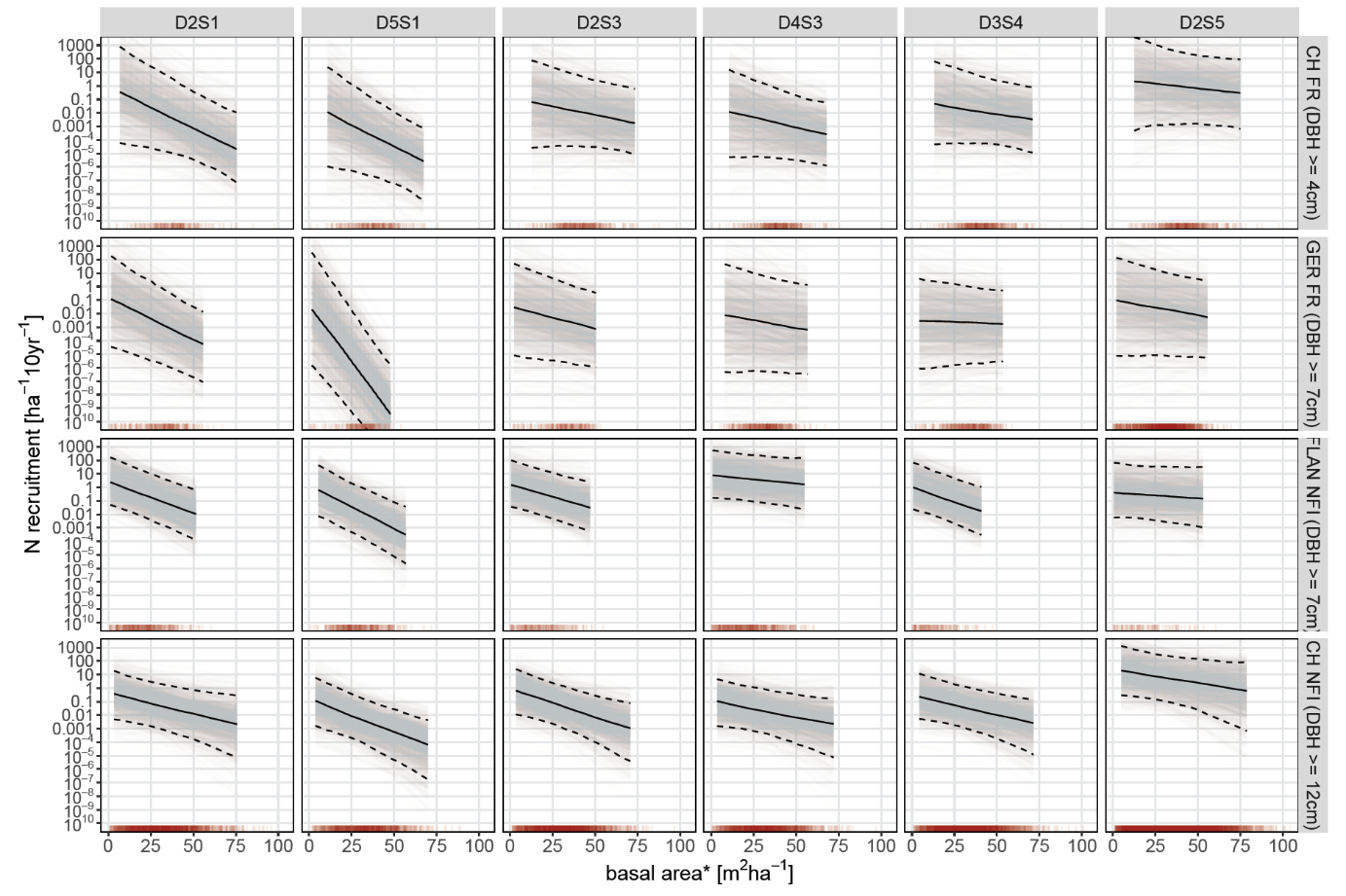


Figure B3: Simulated abundance of tree recruitment given different values of basal area for each data set. Values range from the 1st to the 99th percentile of values where a trait group has been observed (both recruitment and non-recruitment). All other values were set to their mean. Each grey line represents one of 1000 simulations. The solid line represents the median estimate whereas the dashed line represents the 5 % and 95 % credible intervals. Rugs at the bottom axis indicate observations where at least one individual (including non-recruitment) of a certain trait group exists.


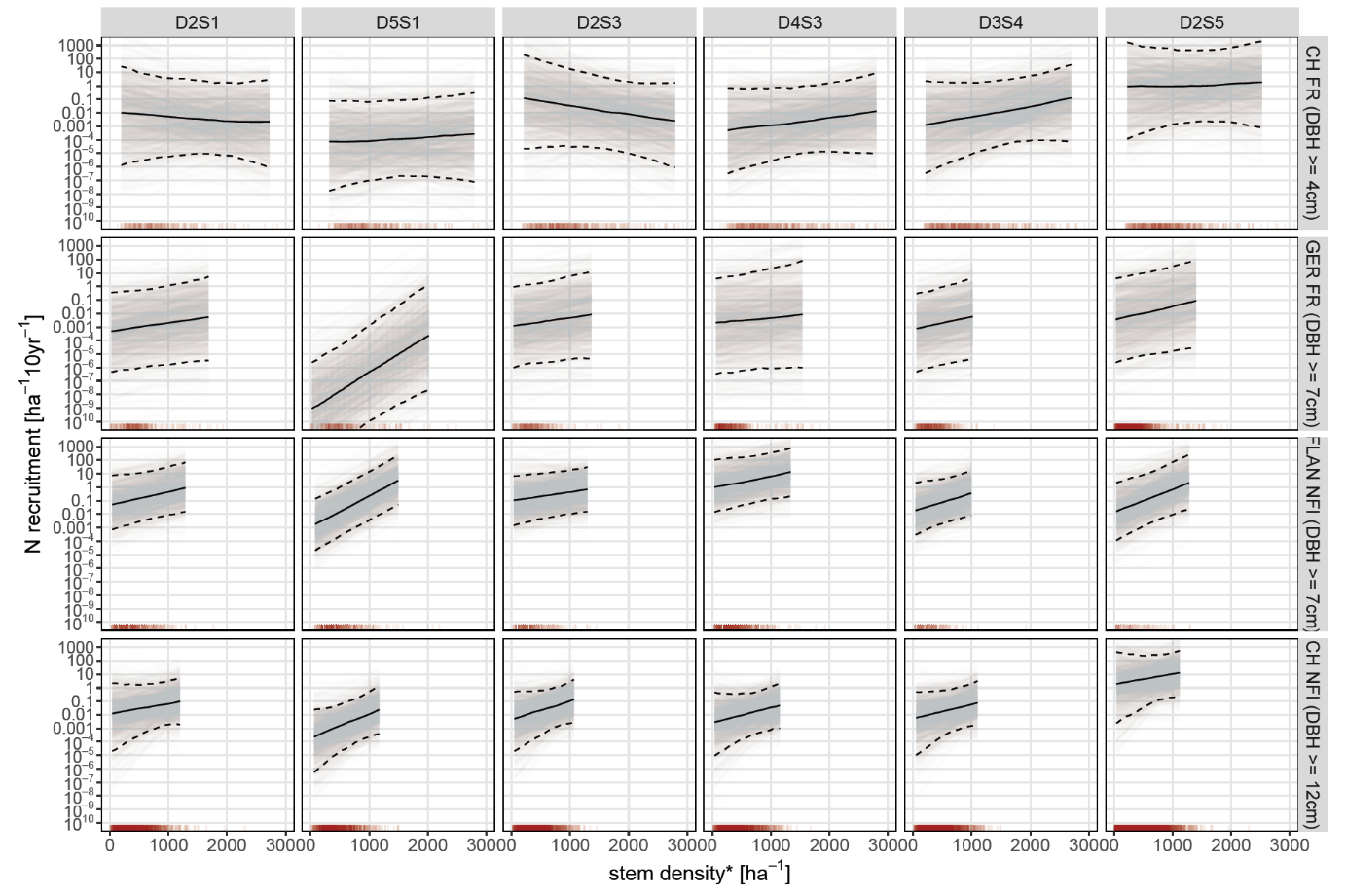
Figure B4: Simulated abundance of tree recruitment given different values of stem density for each data set. Values range from the 1st to the 99th percentile of values where a trait group has been observed (both recruitment and non-recruitment). All other values were set to their mean. Each grey line represents one of 1000 simulations. The solid line represents the median estimate whereas the dashed line represents the 5 % and 95 % credible intervals. Rugs at the bottom axis indicate observations where at least one individual (including non-recruitment) of a certain trait group exists.


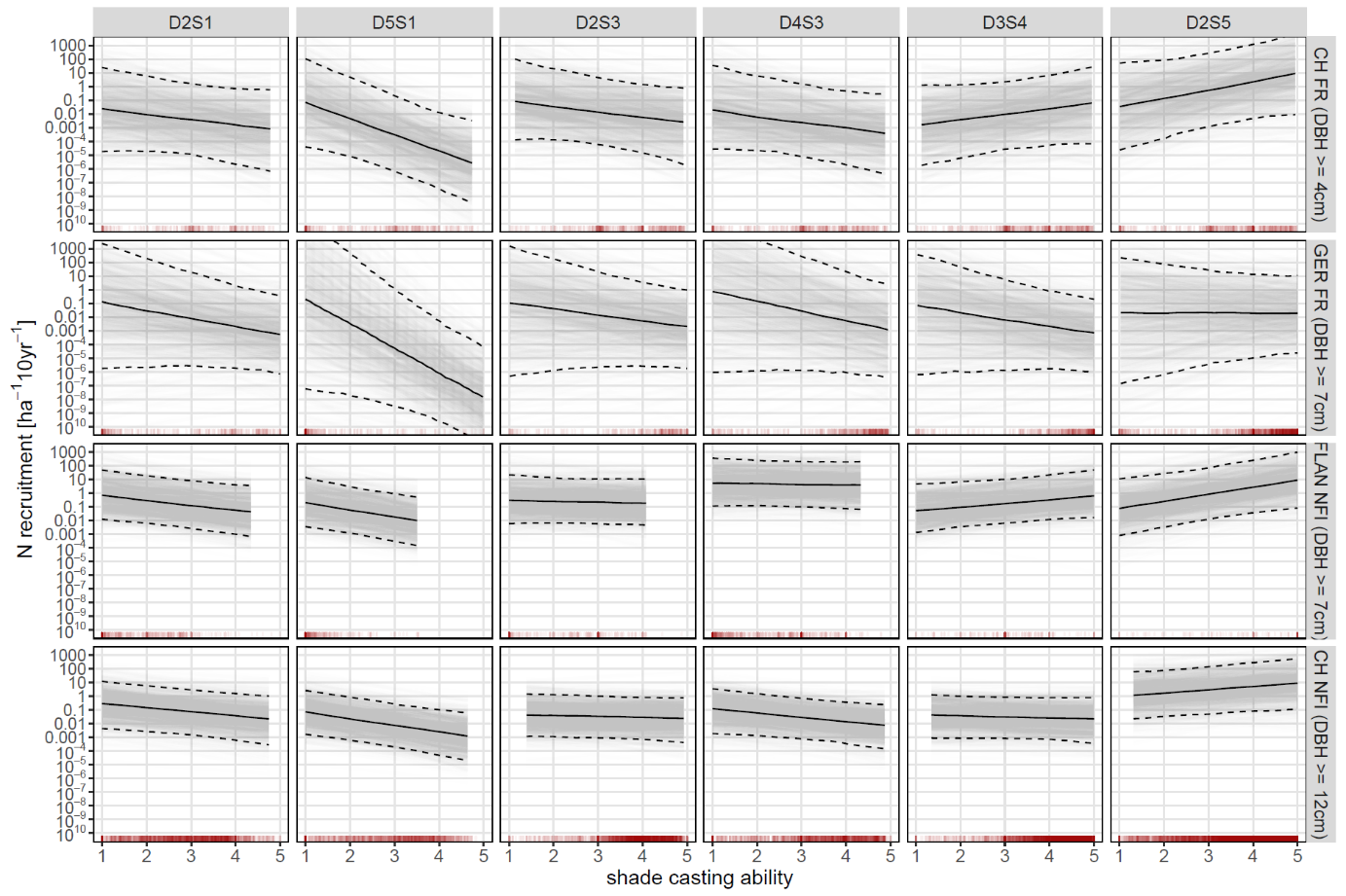
Figure B5: Simulated abundance of tree recruitment given different values of shade casting ability for each data set. Values range from the 1st to the 99th percentile of values where a trait group has been observed (both recruitment and non-recruitment). All other values were set to their mean. Each grey line represents one of 1000 simulations. The solid line represents the median estimate whereas the dashed line represents the 5 % and 95 % credible intervals. Rugs at the bottom axis indicate observations where at least one individual (including non-recruitment) of a certain trait group exists.


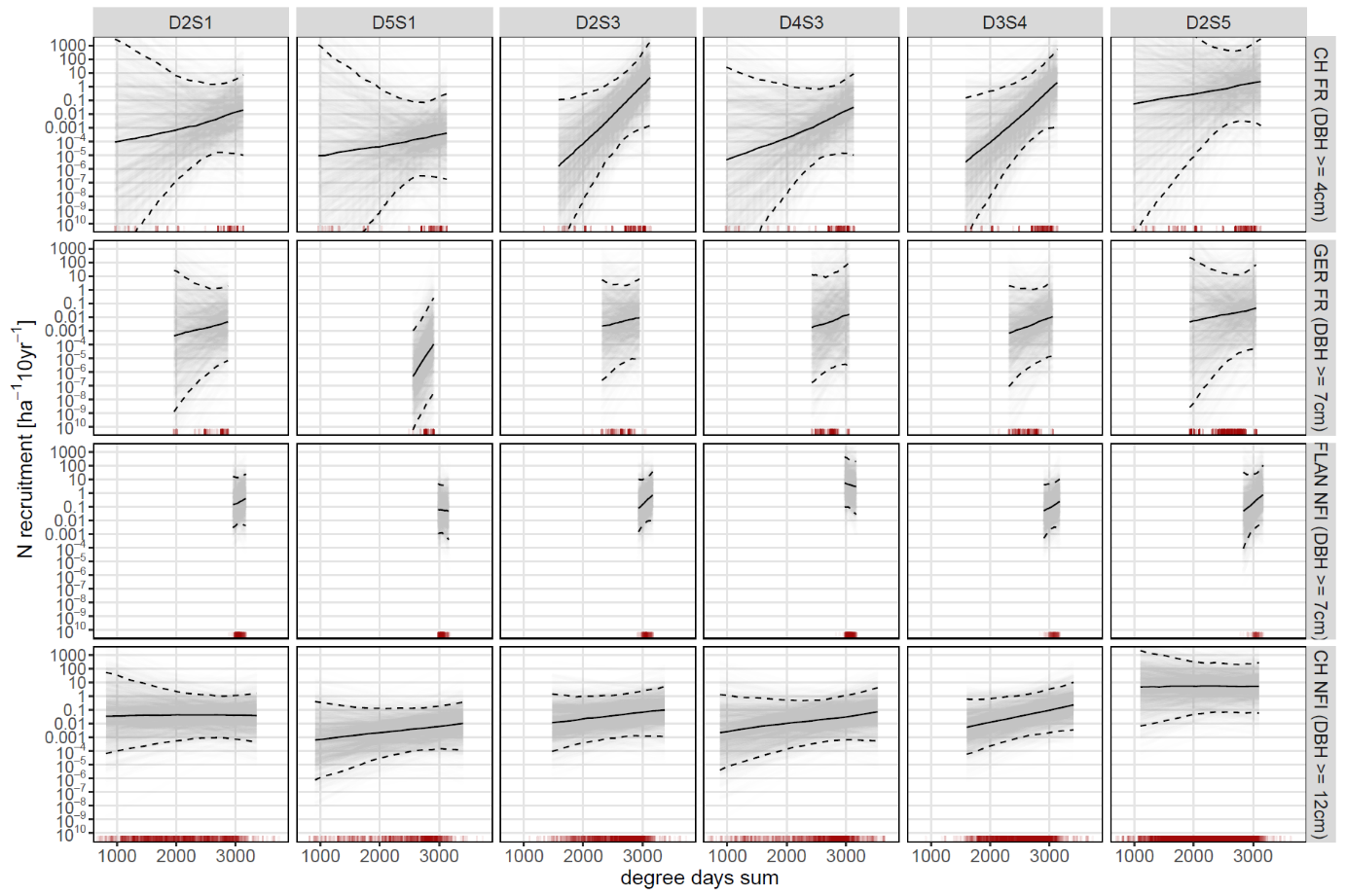
Figure B6: Simulated abundance of tree recruitment given different values of degree days sum for each data set. Values range from the 1st to the 99th percentile of values where a trait group has been observed (both recruitment and non-recruitment). All other values were set to their mean. Each grey line represents one of 1000 simulations. The solid line represents the median estimate whereas the dashed line represents the 5 % and 95 % credible intervals. Rugs at the bottom axis indicate observations where at least one individual (including non-recruitment) of a certain trait group exists.


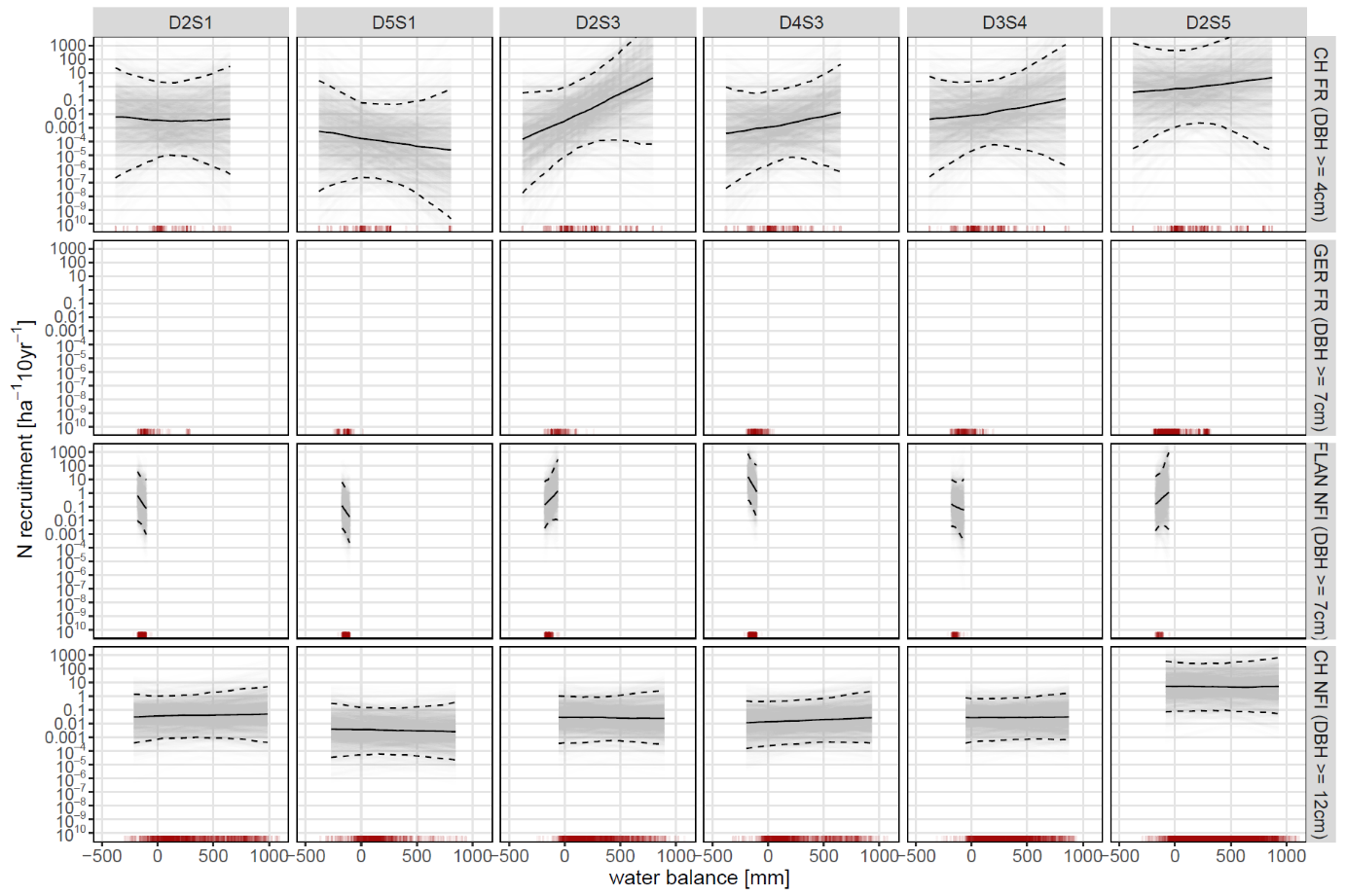
Figure B7: Simulated abundance of tree recruitment given different values of water balance for each data set. Values range from the 1st to the 99th percentile of values where a trait group has been observed (both recruitment and non-recruitment). All other values were set to their mean. Each grey line represents one of 1000 simulations. The solid line represents the median estimate whereas the dashed line represents the 5 % and 95 % credible intervals. Rugs at the bottom axis indicate observations where at least one individual (including non-recruitment) of a certain trait group exists.


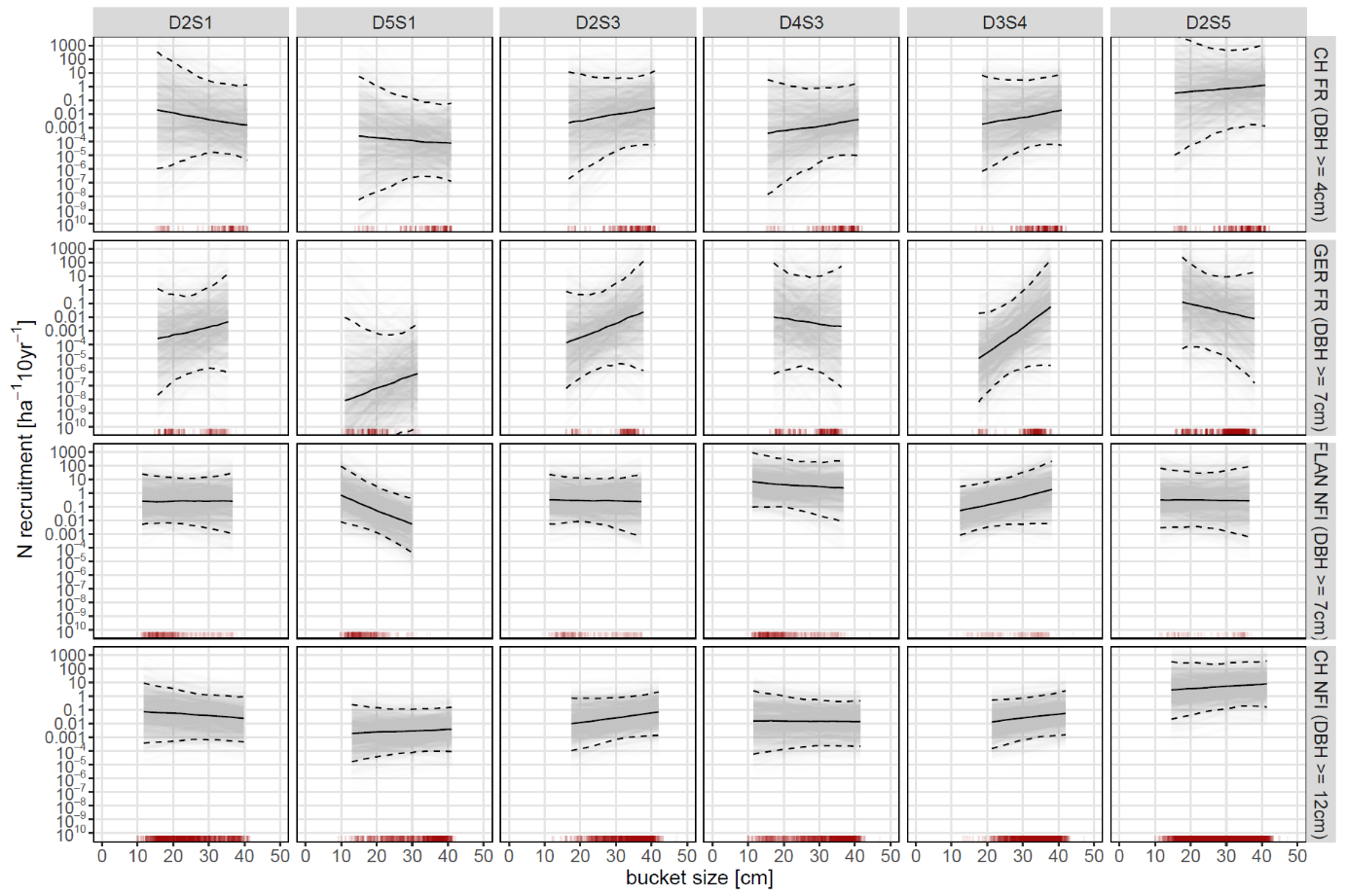
Figure B8: Simulated abundance of tree recruitment given different values of bucket size for each data set. Values range from the 1st to the 99th percentile of values where a trait group has been observed (both recruitment and non-recruitment). All other values were set to their mean. Each grey line represents one of 1000 simulations. The solid line represents the median estimate whereas the dashed line represents the 5 % and 95 % credible intervals. Rugs at the bottom axis indicate observations where at least one individual (including non-recruitment) of a certain trait group exists.


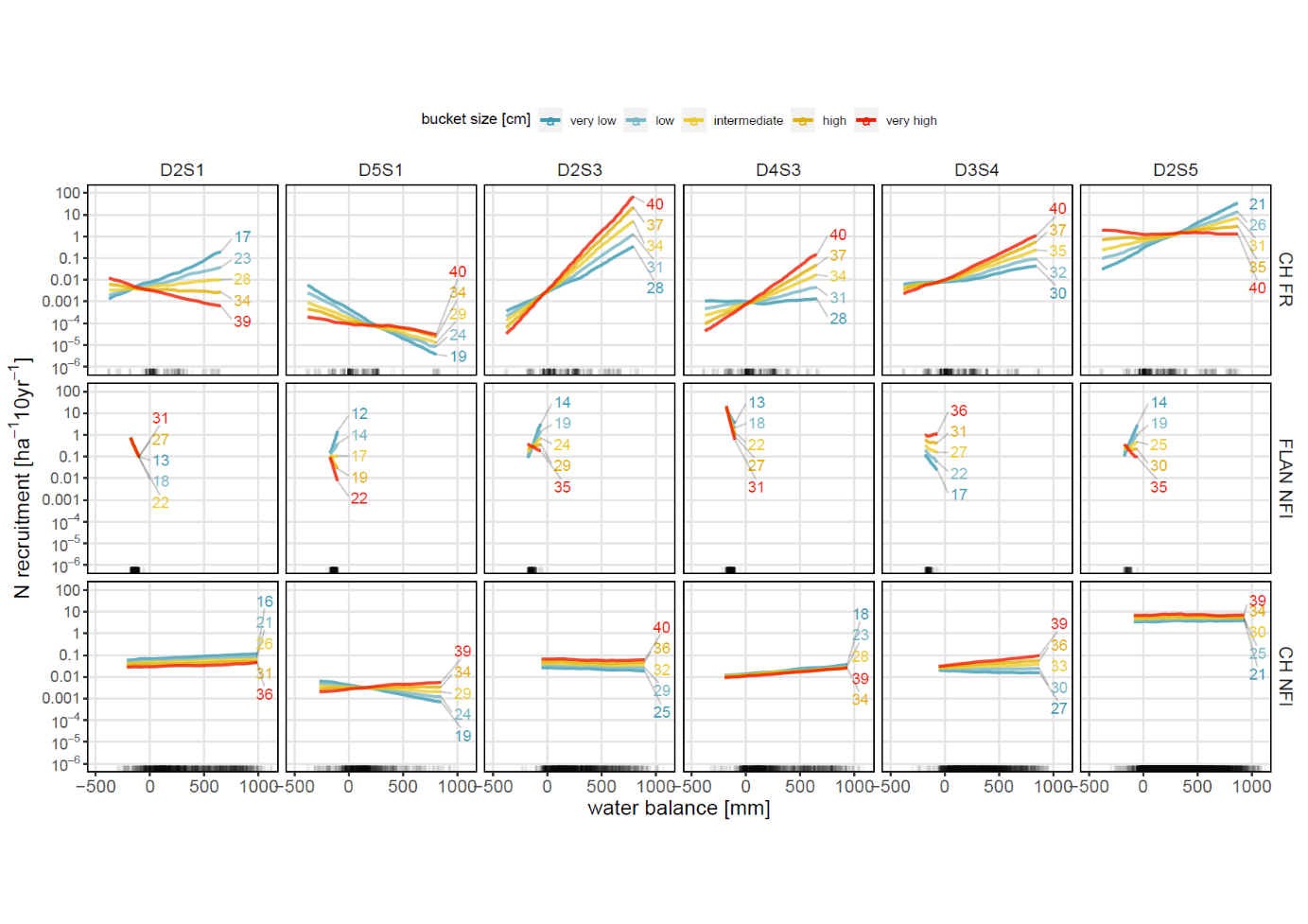
Figure B9: Simulated abundance of tree recruitment given different values of water balance and bucket size for each data set. Values for water balance range from the 1st to the 99th percentile of values where a trait group has been observed (both recruitment and non-recruitment). Bucket size is shown for the 10th (very low), 30th (low), 50th (intermediate), 70th (high) and 90th (very high) percentile. All other values were set to their mean. Each grey line represents one of 1000 simulations. The solid line represents the median estimate whereas the dashed line represents the 5 % and 95 % credible intervals. Rugs at the bottom axis indicate observations where at least one individual (including non-recruitment) of a certain trait group exists.
