# Supplementary material for: Tree recruitment is determined by stand structure and shade tolerance with uncertain role of climate and water relations
Source: Ecol Evol. 2021 Aug 19;11(17):12182–203. doi: 10.1002/ece3.7984 (PMC8427579; doi:10.1002/ece3.7984)
Supplement: Supplementary file 3 — Appendix S3 [file ECE3-11-12182-s002.docx]

Appendix S3

#
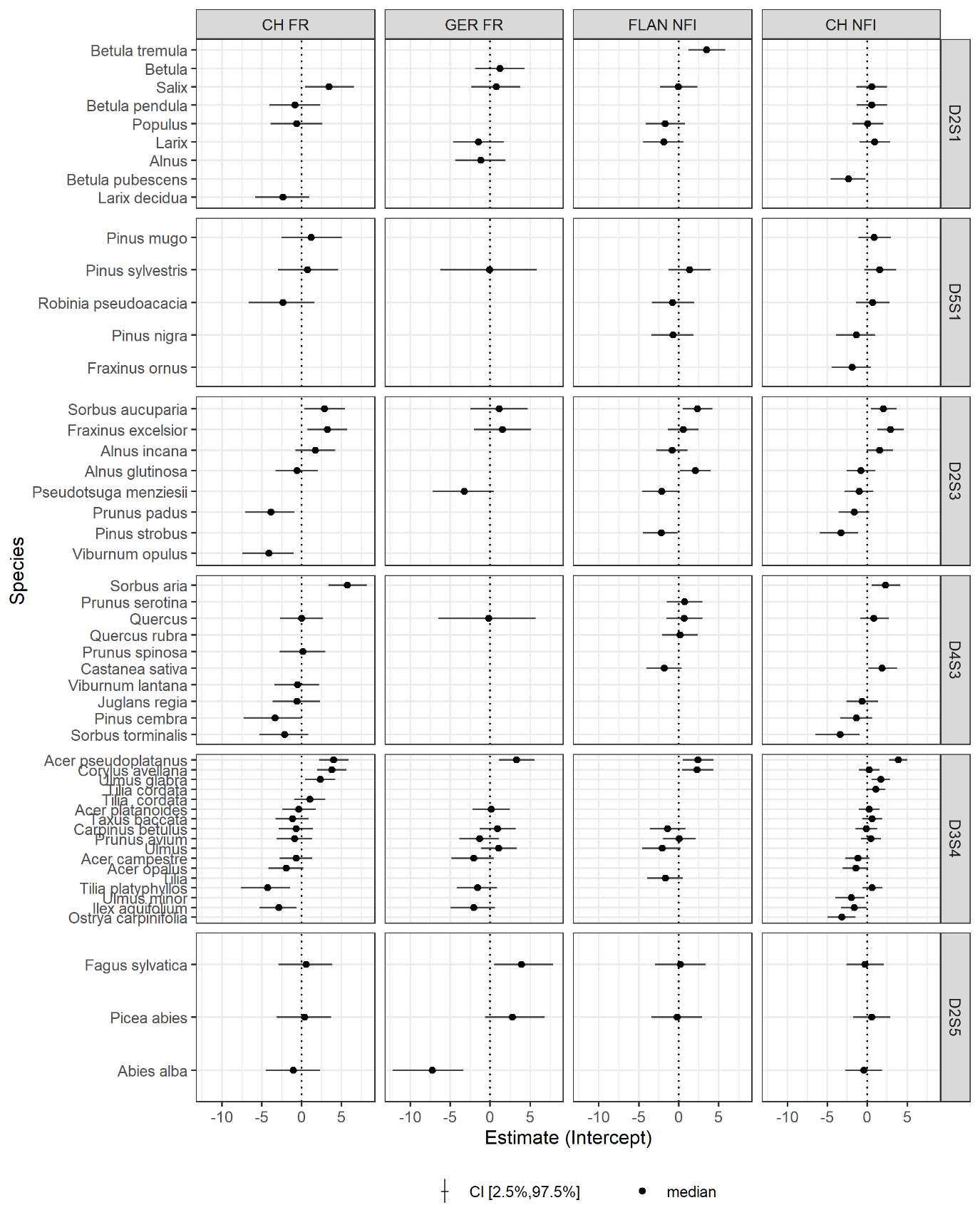
C1 species level random effects

Figure C1: Intercept random effects for each species separated by data set and trait group


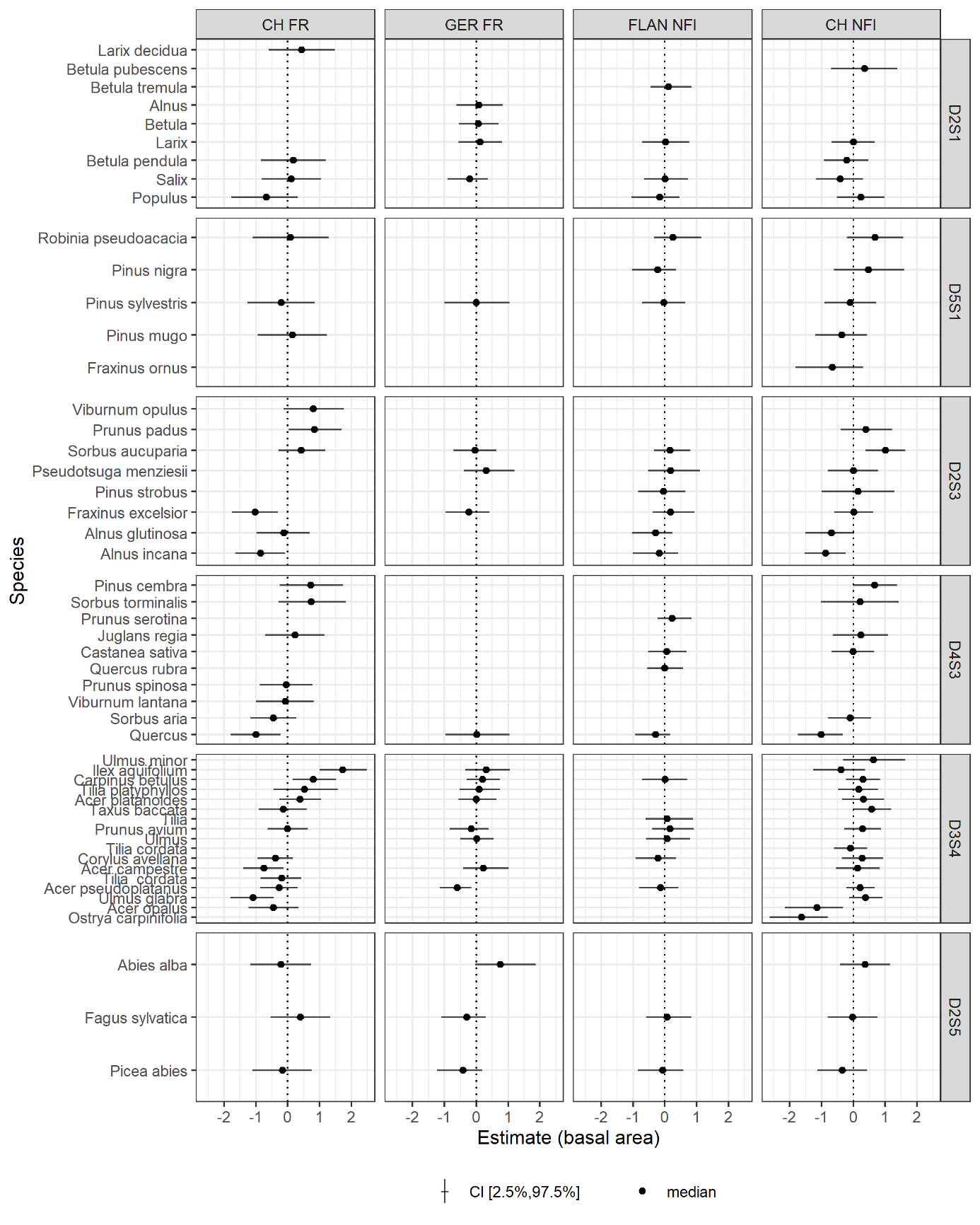
Figure C2: Basal area random effects for each species separated by data set and trait group


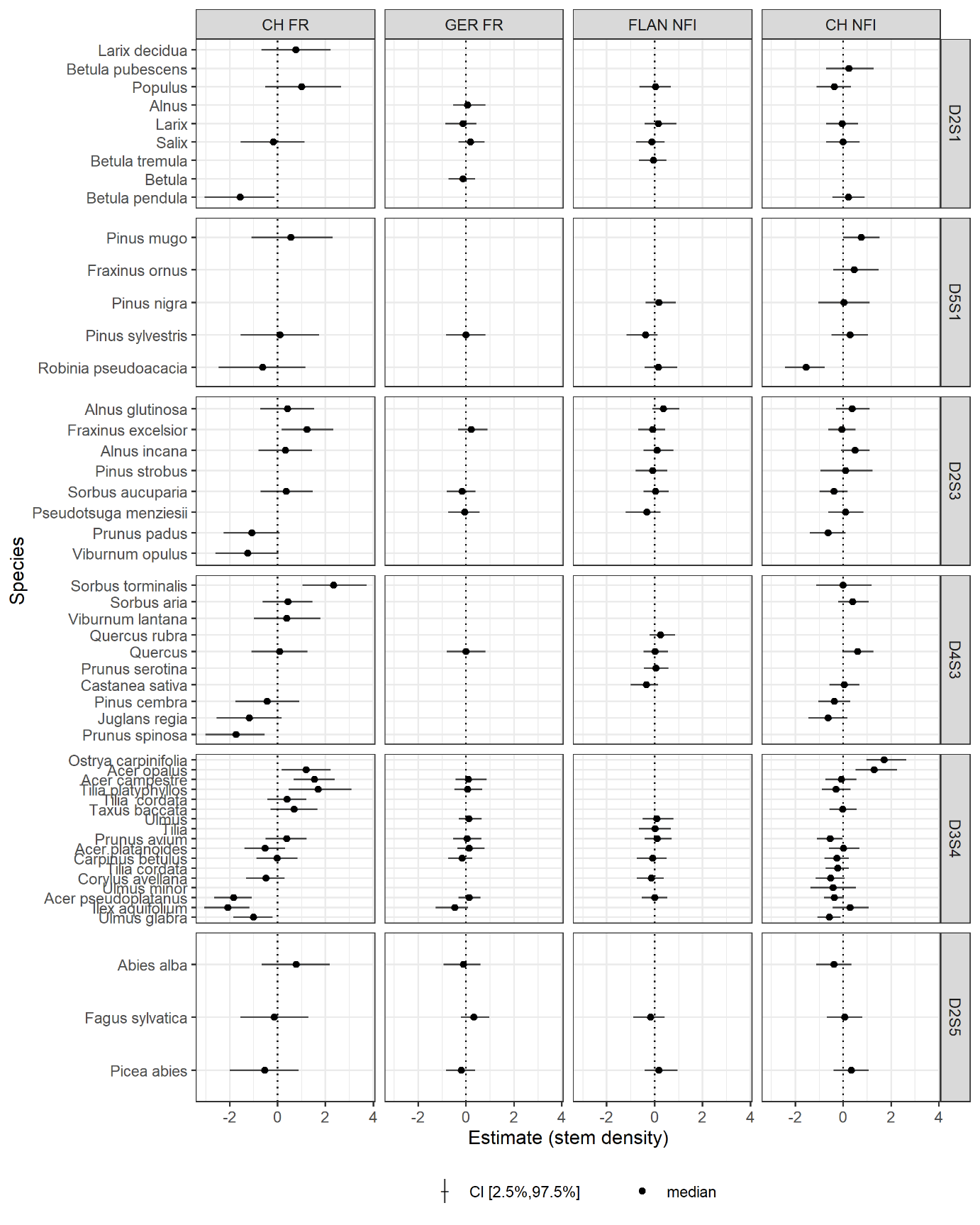
Figure C3: Stem density random effects for each species separated by data set and trait group


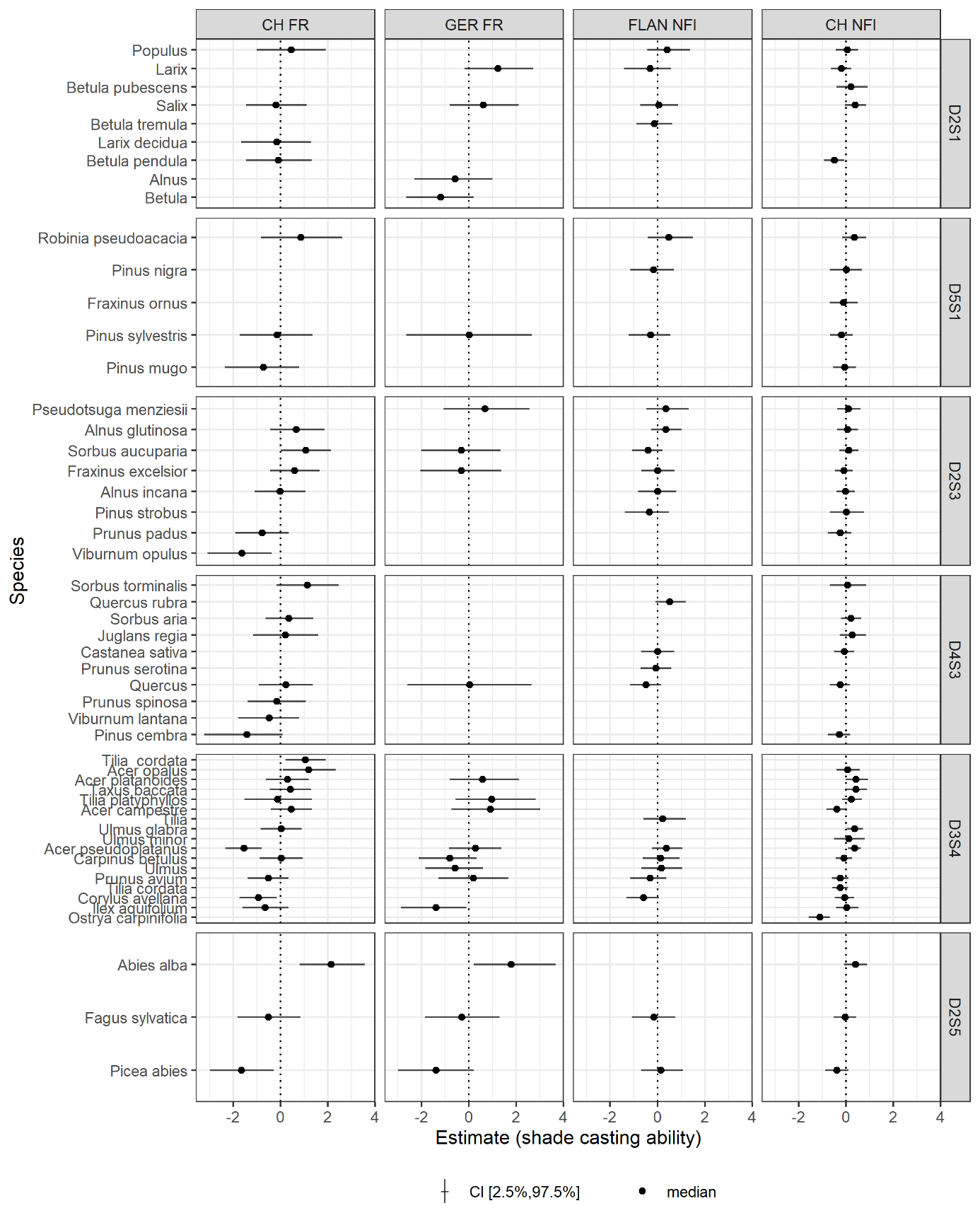
Figure C4: Shade casting ability random effects for each species separated by data set and trait group


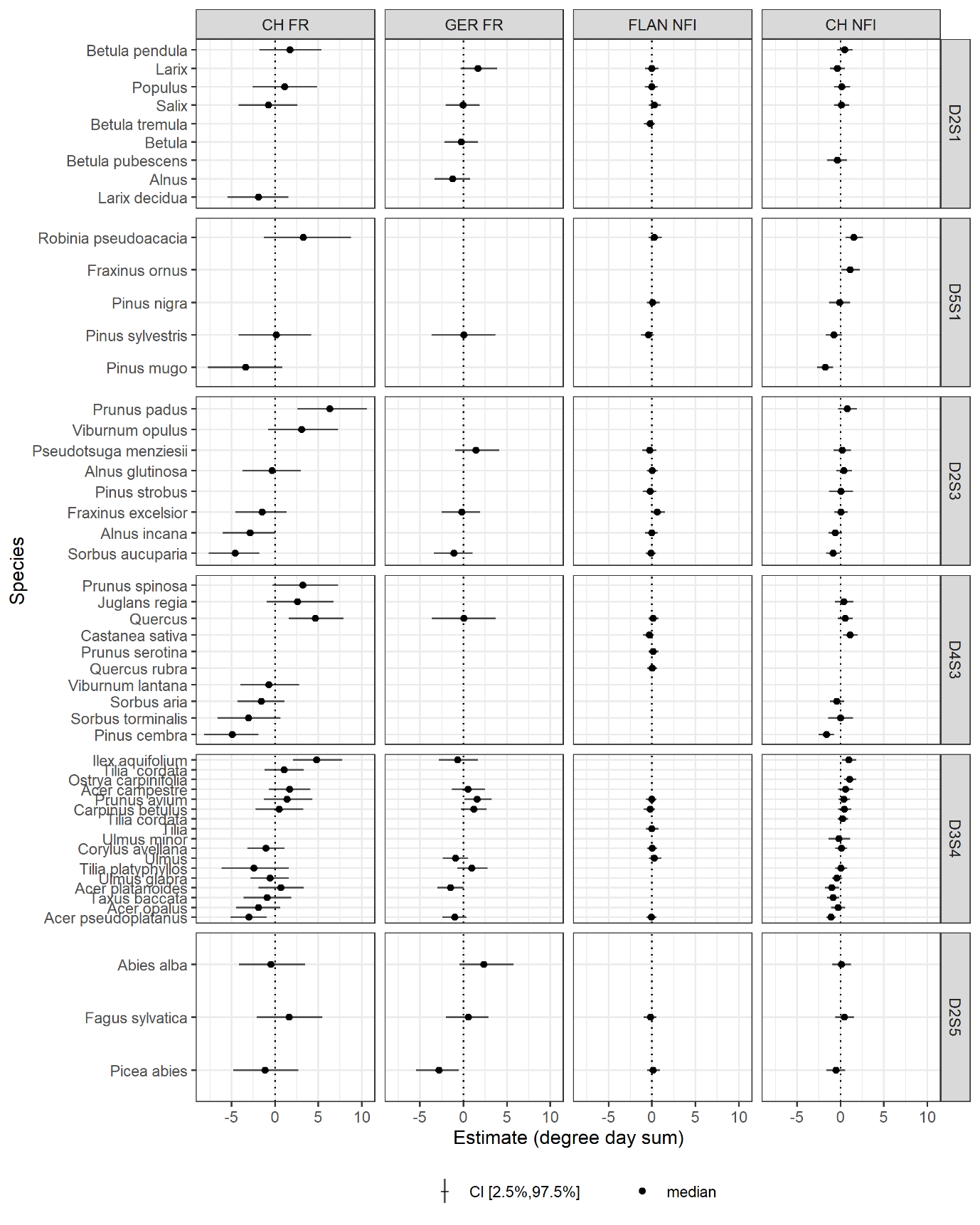
Figure C5: Degree day sum random effects for each species separated by data set and trait group


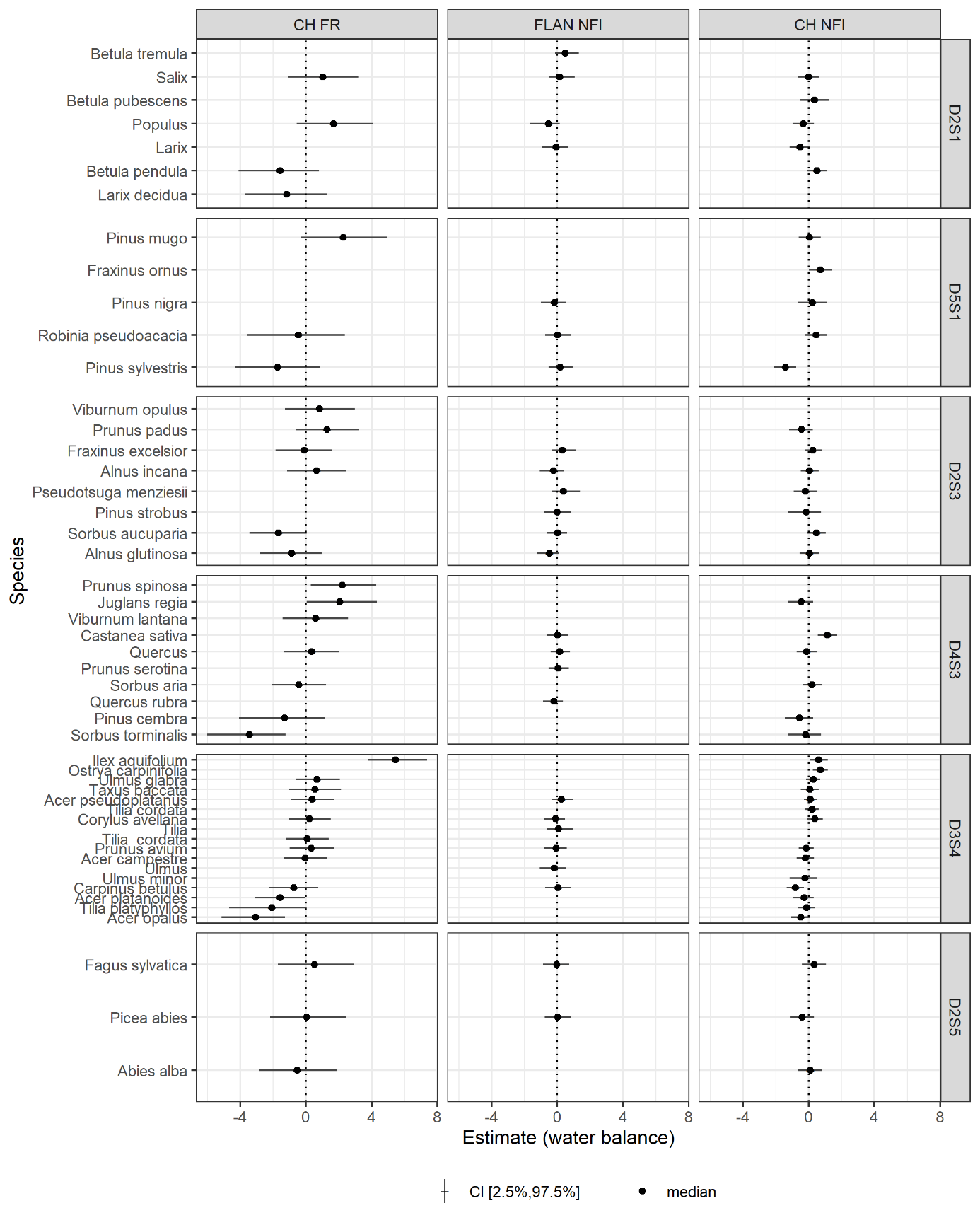
Figure C6: Water balance random effects for each species separated by data set and trait group


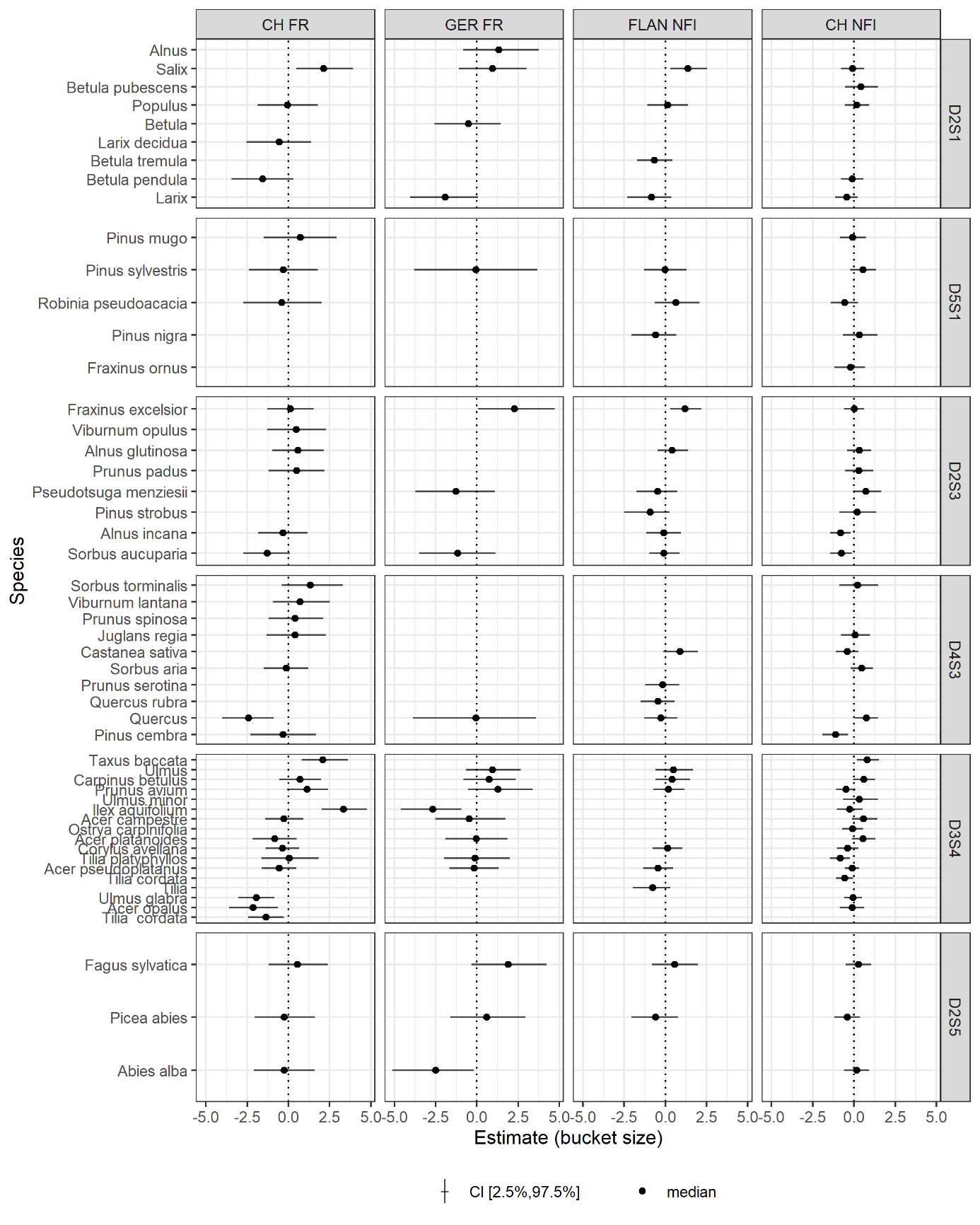
Figure C7: Bucket size random effects for each species separated by data set and trait group


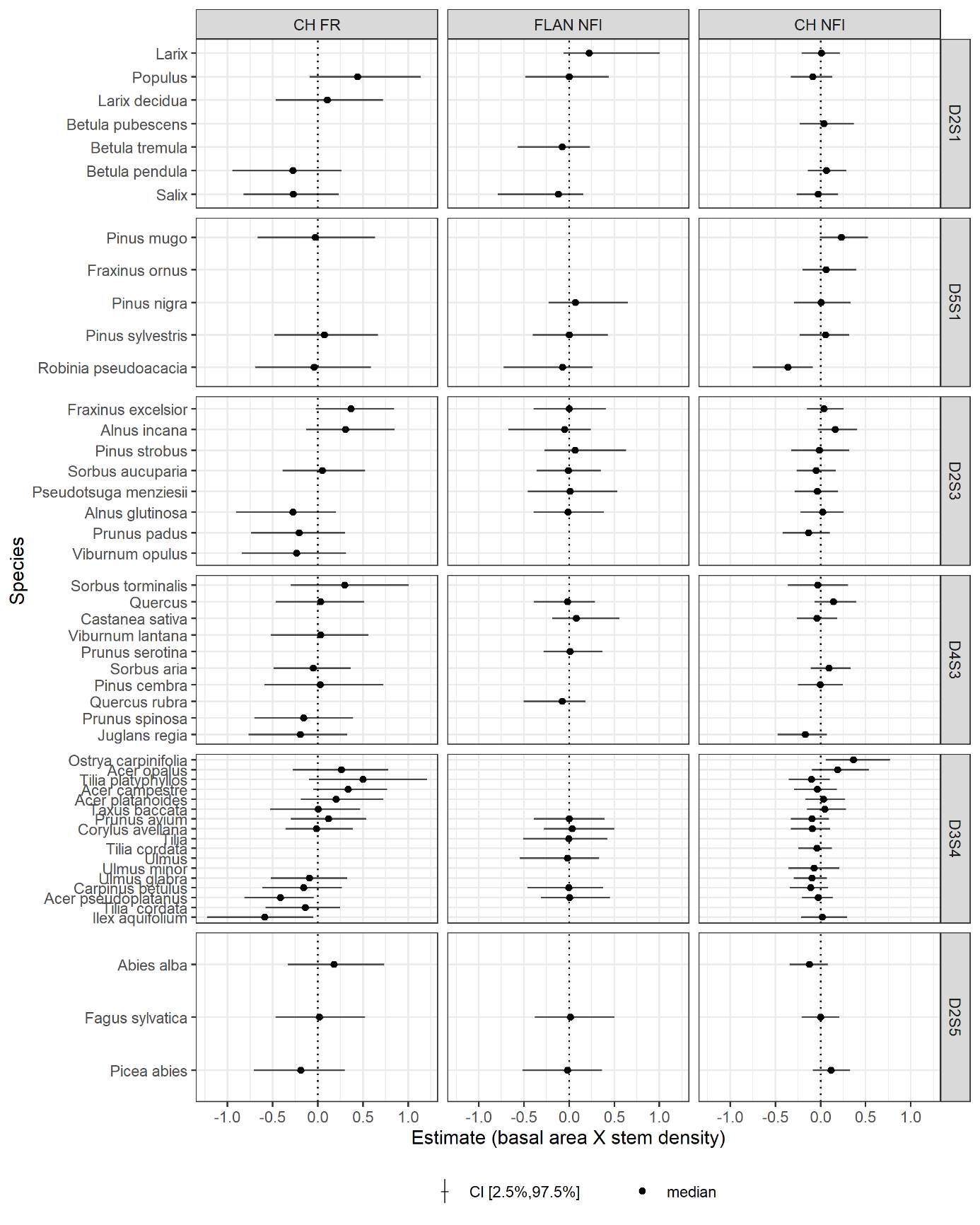
Figure C8: Random effects of interaction between basal area and stem density for each species separated by data set and trait group


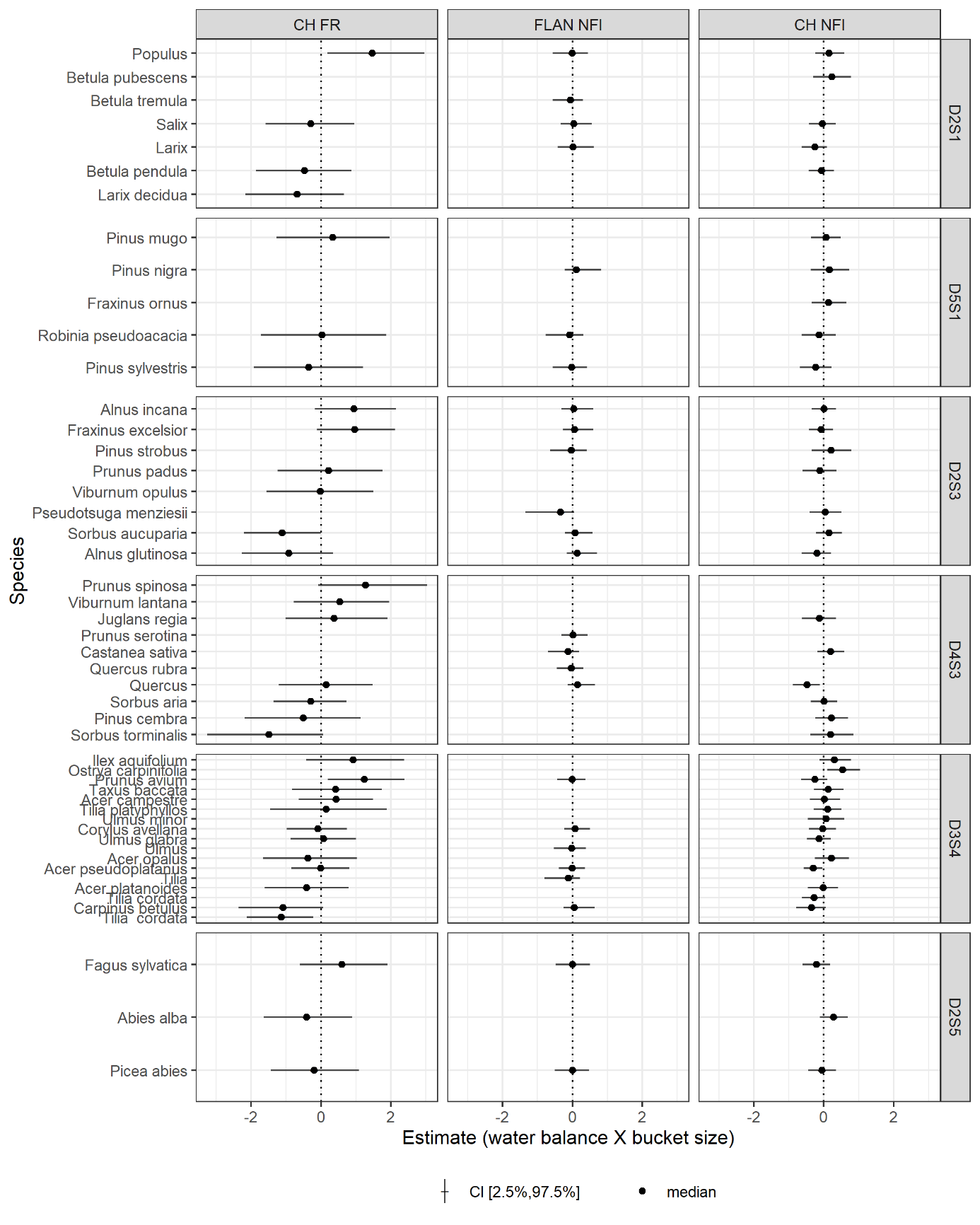
Figure C9: Random effects of interaction between water balance and bucket size for each species separated by data set and trait group
